# Development of artesunate intelligent prodrug liposomes based on mitochondrial targeting strategy

**DOI:** 10.1186/s12951-022-01569-5

**Published:** 2022-08-13

**Authors:** Liwei Gu, Jiaxing Zhang, Dandan Liu, Jiayun Chen, Shuzhi Liu, Qing Peng, Ya Tian, Maobo Du, Junzhe Zhang, Wei Xiao, Shuo Shen, Jigang Wang

**Affiliations:** 1 Artemisinin Research Center and Institute of Chinese Materia Medica, China Academy of Chinese Medical Sciences, Beijing, 100700 People’s Republic of China; 2 School of Traditional Chinese Medicine, Southern Medical University, Guangzhou, 510515 People’s Republic of China; 3 Institute of Basic Medical Sciences of Xiyuan Hospital, China Academy of Chinese Medical Sciences, Beijing, 100091 People’s Republic of China

**Keywords:** Artesunate smart conjugate, Mitochondrial targeting, Liposomes, Anti-tumor, Mitophagy

## Abstract

Breast cancer is the leading cause of cancer-related deaths in women and remains a formidable therapeutic challenge. Mitochondria participate in a myriad of essential cellular processes, such as metabolism, and are becoming an ideal target for cancer therapy. Artemisinin and its derivatives have demonstrated multiple activities in the context of various cancers. Mitochondrial autophagy(mitophagy) is one of the important anti-tumor mechanisms of artemisinin drugs. However, the lack of specific tumor targeting ability limits the anti-tumor efficacy of artemisinin drugs. In this study, a GSH-sensitive artesunate smart conjugate (TPP-SS-ATS) was synthesized and liposomes (TPP-SS-ATS-LS) that target tumor cells and mitochondria were further prepared. The advantages of TPP-SS-ATS-LS targeting to the breast tumor were verified by in vivo and in vitro evaluations. In our study, the cytotoxicity was obviously enhanced in vitro and tumor growth inhibition rate was increased from 37.7% to 56.4% at equivalent artesunate dosage in breast cancer orthotopic implanted mice. Meanwhile, mitochondrial dysfunction, suppression of ATP production and respiratory capacity were detected in breast cancer cells. We further discovered that TPP-SS-ATS-LS inhibited tumor cells proliferation through mitophagy by regulating PHB2 and PINK1 expression_._ These results provide new research strategies for the development of new artemisinin-based anti-tumor drugs.

**Supplementary Information:**

The online version contains supplementary material available at 10.1186/s12951-022-01569-5.

## Introduction

Breast cancer is the most common cancer worldwide among women and the most common type of cancer that results in death. According to the latest report on the global cancer burden in 2020 released by the International Agency for Research on Cancer (IARC) of the World Health Organization (WHO), the number of new cases of breast cancer in 2020 reached 2.26 million, becoming the world's most prevalent cancer type for the first time [1, 2]. The new cases for female breast cancer are the world’s highest [1]. That means one in four cancer patients worldwide suffers from breast cancer, and one in six cancer fatalities is attributable to breast cancer. China is a major country in breast cancer, with about 420,000 new cases and nearly 120,000 deaths in 2020 [3].

In the treatment of breast cancer, chemotherapeutic drugs have relatively serious side effects, and biological anti-tumor drugs are expensive and suffer from low treatment compliance [4, 5]. The disadvantages of these drugs make it difficult for some patients to accept, and this can affect the treatment of the disease. Therefore, the development of a new type of anti-tumor drug with high efficacy and low toxicity is of great significance for the treatment of breast cancer. Artemisinin, as a highly effective antimalarial drug, was discovered by Chinese scientists in the 1970s [6]. This type of drugs (dihydroartemisinin, artesunate and artemether) [7, 8] has a unique oxygen bridge structure, which destroys the cell membrane structure through oxidation and produces a powerful antimalarial effect. Due to the high efficacy and low toxicity of artemisinin drugs, millions of lives of malaria patients have been saved [9]. Likewise, it has been identified that artemisinin drugs can inhibit the proliferation of various tumor cells, including breast cancer, leukemia, hepatic carcinoma and so on [10–13]. The related research has received extensive attention in recent years due to the unique anti-tumor mechanism of artemisinin-based drugs.

Mitochondria play important roles in bioenergetics, ATP synthesis, metabolism, and signaling, and are closely related to many diseases [14]. Mitochondria perform a central role in mitophagy and mitochondrial apoptosis [15]. Mitophagy is the selective degradation of mitochondria by autophagy. It often occurs to defective mitochondria following damage or stress. This is generally regarded as the main mechanism of mitochondrial quality and quantity control. Artesunate is a promising anticancer agent, and multiple anti-tumor mechanisms of artesunate have been reported, including promoting oxidative stress response [16], inducing the apoptosis and autophagy process, activating ferroptosis [17, 18], reducing angiogenesis [19, 20], suppressing metastasis [21], and so on. A recent study demonstrated that artesunate could also trigger mitochondrial autophagy in tumor cells through oxidative effects [22], thereby inducing an anti-tumor effect. However, the exact molecular mechanism is not completely understood until now.

**Scheme 1:**
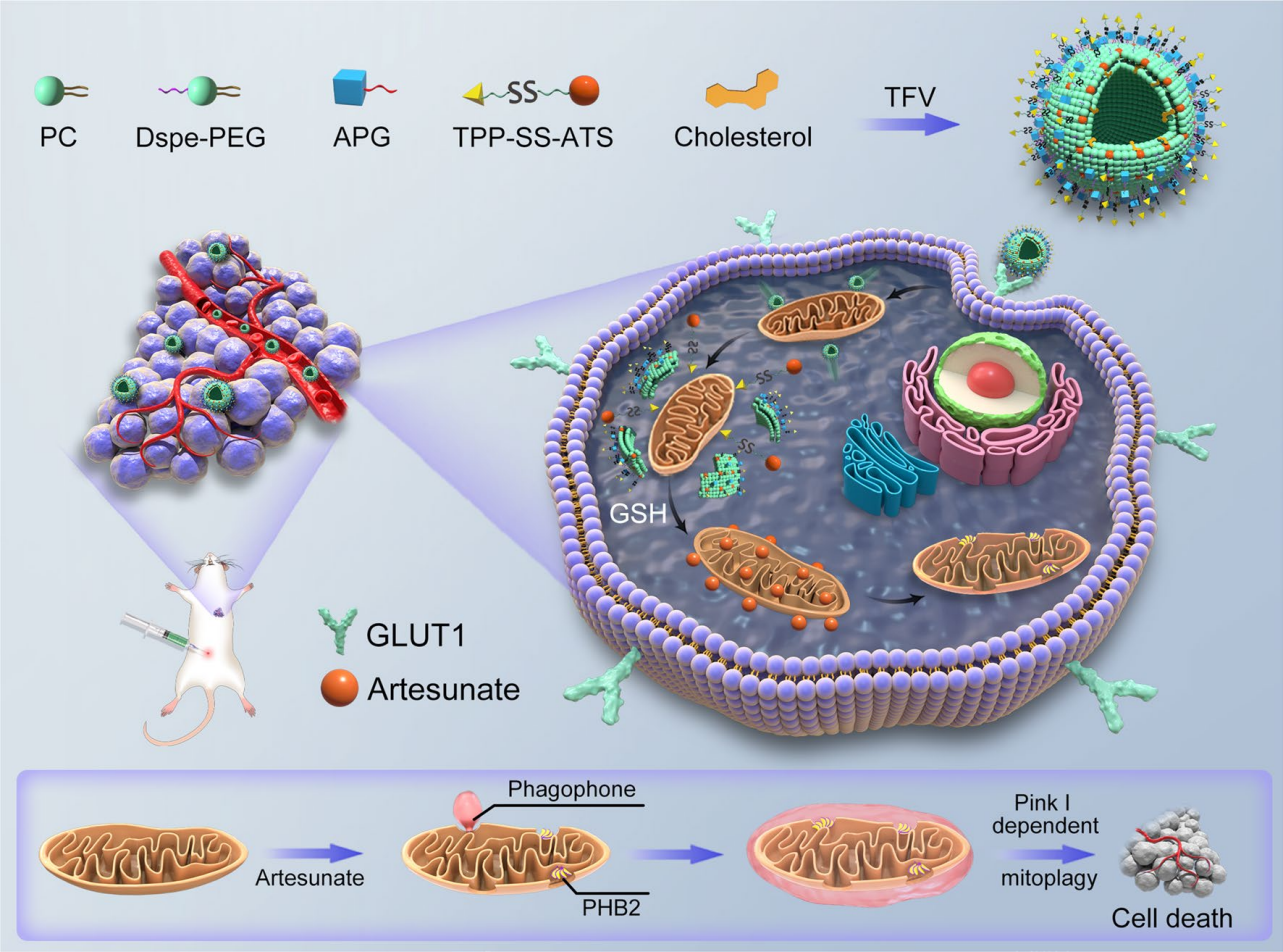
Schematic representation of tumor and mitochondrial dual-targeting artesunate liposomes against breast cancer. In scheme1, APG represented alkyl polyglucoside, and TFV represented thin film vaporation and dispersion method.

Because artemisinin-based drugs lack the ability to target tumor cells (and other optimal organelles), their effectiveness as anti-tumor drugs remain limited. In order to further improve the anti-tumor effect of artemisinin-based drugs, we have developed artesunate anti-tumor liposomes (TPP-SS-ATS-LS) with dual targeting of tumor cells and tumor cell mitochondria based on previous research on targeted anti-tumor liposomes. GLUT1 was highly expressed in tumor cells and it was used as the target in the study. PEGylation and targeted molecule with glucose fragment were modified on liposomes, so as to prolong the circulation time of liposomes and improve the tumor targeting of liposomes. To achieve mitochondrial targeting [16], triphenylphosphine was used as the targeting head and the target molecule was connected with artesunate through disulfide bond to form artesunate mitochondrial targeting intelligent molecule (TPP-SS-ATS) [23]. In the preparation of anti-tumor liposomes, the smart molecule can be modified in the liposome membrane. The mitochondrial targeting effect [24–26] of TPP was used to attract liposomes around the mitochondria. After TPP-SS-ATS was released around the mitochondria, the disulfide bond of TPP-SS-ATS was snapped to release artesunate (ATS) under the high glutathione (GSH) environment of the tumor cell [27–29]. The mitochondrial targeted delivery and intelligent release of artesunate could be realized based on the above research ideas (Scheme 1).

In this paper, technical methods such as transmission electron microscopy (TEM), differential scanning calorimetry (DSC) and molecular dynamics simulation were used to systematically evaluate the nanopharmaceutical characteristics of TPP-SS-ATS-LS. In breast cancer as a disease model, the advantages of TPP-SS-ATS-LS against breast tumor were verified by in vivo and in vitro evaluations. Further studies on anti-tumor mechanisms have shown that the novel nano-preparation inhibits tumor cell proliferation through the mitochondrial autophagy pathway, which is mediated by down-regulating PHB2 and PINK1 expression [30]. The relevant research conclusions of this paper provide new research ideas for the development of new artemisinin-based anti-breast tumor drugs.

## Methods and materials

### Materials

Artesunate (ATS) was purchased from Kunyao Group Chongqing Wulingshan Pharmaceutical Co., Ltd. (Chongqing, China). Soybean lecithin (PC95), cholesterol and DSPE-mPEG2000 were purchased from A.V.T. (Shanghai) Pharmaceutical Co., Ltd. (Shanghai, China). FITC-PEG-DSPE (MW 2000) was purchased from Shanghai Pengshuo Biotechnology Co., Ltd. (Shanghai, China). DiR iodide was purchased from AAT Bioquest, Inc. (USA). Dichloromethane and Methanol were purchased from Shanghai TITAN Technology Co., Ltd. (Shanghai, China). (4-carboxybutyl) triphenylphosphonium bromide (TPP), *N*,*N*-dicyclohexylcarbodiimide (DCC), 3-(3-dimethylaminopropyl)-1-ethylcarbodiimide hydrochloride (EDCI), glutathione and 4-dimethylaminopyridine (DMAP) were purchased from Adamas Reagent Co., Ltd. (Shanghai, China). 2,2-dithiodiethanol was purchased from Tokyo Chemical Industry Co., Ltd.. Acetonitrile HPLC grade was purchased from Thermo Fisher Scientific, Inc. (Massachusetts, USA). Purified water was purchased from A.S. Watson Group Ltd. (Hong Kong, China). Centrifugal ultrafiltration tubes were purchased from Merk Millipore Ltd. (Darmstadt, Germany). HPLC chromatographic column (Luna C18(2), 4.6 × 100 mm, 3 μm) was purchased from Phenomenex (USA). The gemcitabine was supplied by Shanghai Titan Scientific Co. Ltd. with a purity of 99%. RPMI1640, Dulbeccoʼs modified Eagle medium (DMEM; Corning, USA), and fetal bovine serum (FBS) were from Corning (Corning, USA). 0.25% trypsin and 100 × penicillin–streptomycin (PS) were purchase from Gibco (USA). Cell Counting Kit-8(CCK-8) is from DOJINDO (Japan). Mito Tracker Red CMXros (M9940) and Hoechst 33342 (C0031) were supplied by Beijing Solarbio Science & Technology (China). Primary antibodies used in this study: LC3(Cat# 14600-1-AP), PINK1 (Cat# 23274-1-AP) and PHB2(12295-1-AP) were supplied by Proteintech, Inc. (Proteintech Group, USA), and β-actin and GAPDH were from Affinity Biosciences (USA).

### Cell lines and animals

Human breast carcinoma cells MCF-7 and MDA-MB-231, and mouse breast carcinoma cells 4T1 were purchased from the Institute of Basic Medical Sciences of Chinese Academy of Medical Sciences (Beijing, China). MDA-MB-231 and 4T1 cells were cultured with RPMI-1640 10%FBS and MCF-7 cells were cultured with DMEM 10%FBS. Female specific-pathogen-free BALB/c mice (aged 4–6 weeks) were purchased from Speifu (Beijing) biotechnology co., LTD (Beijing, China). The experiments were housed in groups of four per cage at a temperature of 23 ± 1 ℃, on a 12 h light–dark cycle, and without restriction to food and water. All experiments were carried out to minimize the number and suffering of animals. The animals were used following the National Research Council's Guide for the Care and Use of Laboratory Animals act “Laboratory Animal Administration Rules” on the use and care of laboratory animals. All experiments were approved by the Animal Ethical and Welfare Committee.

### Synthesis and structural characterization of the GSH-sensitive artesunate smart conjugate TPP-SS-ATS

**Fig. 1:**
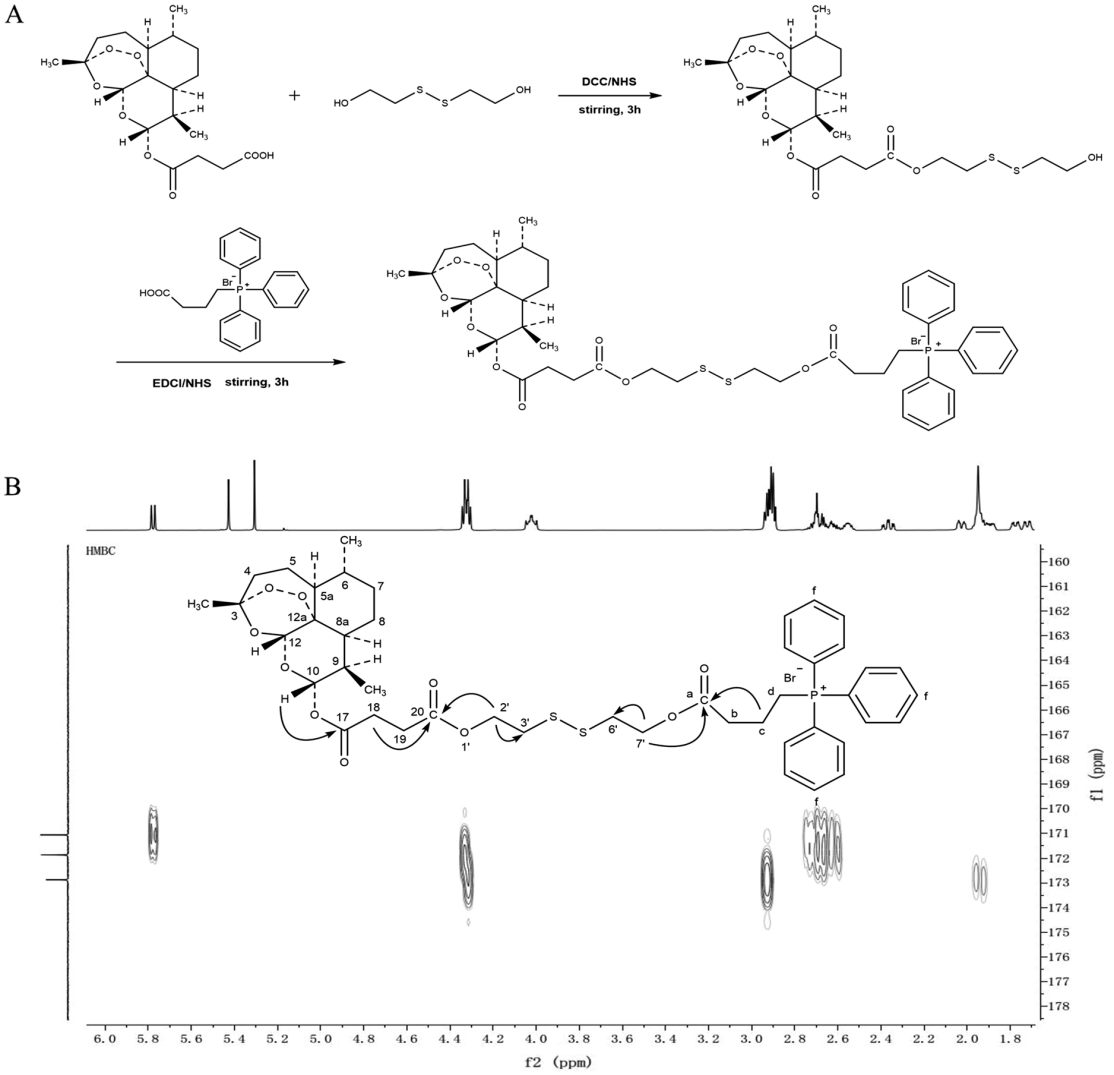
**A** Schematic illustration of synthesis for TPP-SS-ATS and chemical structures of SS-ATS and TPP-SS-ATS. **B** HMBC spectrum of TPP-SS-ATS

1. Synthesis of chemical intermediate (SS-ATS): the schematic synthetic method of SS-ATS is shown in Fig. 1A. Artesunate 5 g and DCC 3.6 g were dissolved in 500 ml of CH_2_Cl_2_ with stirring at 350 rpm for 1 h at room temperature, then 2,2-dithiodiethanol 3.0 g and DMAP 0.5 g were added into the CH_2_Cl_2_ solution. The reaction was carried out for 3 h at room temperature. After that, the CH_2_Cl_2_ solvent was recovered at 35 ℃ by rotary evaporator to obtain the crude SS-ATS product. The reaction mixture was purified by silica gel (200–300 mesh) column chromatography with gradient mobile phase of petroleum ether: ethyl acetate (10:1 to 2:1) and the purified SS-ATS was obtained. The structure of the SS-ATS was determined by AVANCE III HD 600 MHz NMR spectroscopy (Bruker, Germany) and API 4000QTRAP MS (Applied Biosystem, USA). (2) Synthesis of TPP-SS-ATS: the schematic synthetic method of TPP-SS-ATS is shown in Fig. 1A. SS-ATS 1.6 g and EDCI 3.2 g were dissolved in 50 ml of CH_2_Cl_2_ with stirring at 350 rpm for 1 h at room temperature, and then TPP 3.2 g and DMAP 0.6 g were added into the CH_2_Cl_2_ solution while stirring. The reaction was carried out for 12 h at room temperature. After the reaction finished, the CH_2_Cl_2_ solvent was recovered at 35 ℃ to obtain the crude TPP-SS-ATS product. The reaction mixture was purified by silica gel (200–300 mesh) column chromatography with gradient mobile phase of CH_2_Cl_2_: methanol (500:1 to 50:1) and the purified TPP-SS-ATS was obtained. The structure of the TPP-SS-ATS was determined by AVANCE III HD 600 NMR spectroscopy and API 4000QTRAP MS.

### Purity check of TPP-SS-ATS

The analytical process was performed by Waters ACQUITY Arc system (Waters, USA). HPLC conditions were as follows. Column: Luna C18(2) column (4.6 × 100 mm, 3 μm); column temperature: 30 ℃; detection wavelength: 267 nm; mobile phase flow rate: 1.0 mL min^−1^; mobile phase: methanol and 0.1% phosphoric acid/water were used as the mobile phase, gradient conditions: linear gradient 40–90% methanol for 30 min. The purity of TPP-SS-ATS was determined by HPLC peak area normalization.

### Preparation of drug-loaded liposomes

#### Preparation of TPP-SS-ATS liposomes

The liposomes were prepared by thin film dispersion method. The specific method was as follows: The soybean lecithin (71.5%), *n*-octyl-β-d-glucoside (7.0%), TPP-SS-ATS (9.5%), cholesterol (3.5%) and DSPE-mPEG2000 (8.5%) were dissolved in dichloromethane (the ratios of solid excipients and drug were weight percentages, the total weight of solid excipients and drug was 100%); the solution was transferred to rotary evaporator bottle, dichloromethane was completely evaporated at 40 °C by decompression; and then the proper amount of water was added into rotary evaporator bottle and sample was hydrated at 150 rpm for 25 min to obtain crude liposomes. The liposomes were under ultrasonic concussion at 250 W for 3 min by SCIENTZ-950E Ultrasonic Cell Crusher (NINGBO SCIENTZ BIOTECHNOLOGY CO., LTD., China) and the TPP-SS-ATS liposomes were obtained after filtration with 0.22 µm microporous membrane. The liposomes were appropriately diluted with normal saline for other experimental studies.

#### Preparation of FITC-TPP-SS-ATS liposomes

The liposomes were prepared by thin film dispersion method. The specific method was as follows: The soybean lecithin (71.5%), *n*-octyl-β-d-glucoside (7.0%), TPP-SS-ATS (9.5%), cholesterol (3.5%), DSPE-mPEG2000 (5.4%) and FITC-PEG-DSPE (3.1%) were dissolved in dichloromethane (the ratios of excipients and drug were weight percentages). Other preparation methods were the same as “Preparation of TPP-SS-ATS liposomes”. The FITC-TPP-SS-ATS liposomes were appropriately diluted with normal saline for in vitro tumor targeting evaluation.

#### Preparation of DiR-TPP-SS-ATS liposomes

The liposomes were prepared by thin film dispersion method. The specific method was as follows: The DiR (0.5%), soybean lecithin (71.0%), *n*-octyl-β-d-glucoside (7.0%), TPP-SS-ATS (9.5%), cholesterol (3.5%) and DSPE-mPEG2000 (8.5%) were dissolved in dichloromethane (the ratios of excipients and drug were weight percentages). Other preparation methods were the same as “Preparation of TPP-SS-ATS liposomes”. The DiR-TPP-SS-ATS liposomes were appropriately diluted with normal saline for in vivo tumor targeting evaluation.

### Preparations of Blank liposomes

The preparation method of blank liposomes was almost the same as that of TPP-SS-ATS liposomes (Preparation of TPP-SS-ATS liposomes). The only difference between them was that the blank liposomes did not contain TPP-SS-ATS in prescription.

### Particle size, zeta potential and polydispersity index (PDI)

The Particle size, zeta potential and PDI of TPP-SS-ATS-LS were measured by a Malvern Zetasizer Nano ZS (Malvern Instruments, UK). All measurements were carried out at 25 ℃. Each measurement was performed in triplicate and the average of the relevant data was used for result analysis.

### Liposomes morphology

TPP-SS-ATS-LS were diluted 5 times with purified water. The diluent was added on 200-mesh copper grid of the electron microscope and the excess sample was removed with a filter paper. After natural drying, the TPP-SS-ATS-LS morphology was observed by a H7650 transmission electron microscope (TEM) (HITACHI, Japan).

### Differential scanning calorimetry (DSC)

The phase transition temperatures of TPP-SS-ATS-LS and Blank LS were measured by a Nano DSC (TA, USA). The temperature scanning range of the samples was 20–100 ℃, and the heating rate was 1 ℃/min. The pressure of sample cell during the experiment was 3 atmospheres. The reference solution was purified water.

### Entrapment efficiency (EE) of liposomes

The encapsulation efficiency (EE) of TPP-SS-ATS-LS was determined by ultrafiltration method. (1) Determination of the total content of TPP-SS-ATS in the liposomes solution (M1): The TPP-SS-ATS-LS were added into the volumetric flask and the TPP-SS-ATS was extracted by ultrasonic extraction with extraction solvent for 15 min at room temperature. The content (M1) of TPP-SS-ATS was determined by Waters ACQUITY Arc system (Waters, USA). (2) Determination of free TPP-SS-ATS content (M2): An appropriate amount of TPP-SS-ATS-LS was added into the ultrafiltration tube (millipore Amicon ultra 4 ml 10 kDa). The ultrafiltration tube was centrifuged at 5000 rpm at 4 ℃ for 120 min to obtain ultrafiltration filtrate and the content (M2) of free TPP-SS-ATS was determined by Waters ACQUITY Arc system (Waters, USA). The average of three parallel experimental data was used to analyze the results. The EE was calculated by the following equation:

$$EE(\% ) = \frac{{M_{1} - M_{2} }}{{M_{1} }} \times 100\% .$$

#### In vitro release study

The artesunate release from TPP-SS-ATS-LS were characterized by the dalysis method. The operation method was as follows: TPP-SS-ATS-LS was added into a 4 cm long dialysis bag (MWCO: 7000–15,000 Da, width: 25 mm). The bag was put into a triangular bottle, which was immersed in 100 ml water or 100 ml 10 mM GSH solution. The in vitro release studies were performed with SKY-100C shaker (Shanghai Sukun Industrial Co., Ltd). Dialysis samples were shaken at 90 rpm at room temperature. Samples were taken at different time points, and the release of artesunate was determined by E2695-2998 HPLC system (Waters, USA). HPLC conditions were as follows. Column: Phenomenex Luna C18(2) column (4.6 × 100 mm, 3 μm); column temperature: 30 ℃; detection wavelength: 216 nm; mobile phase flow rate: 1.0 mL min^−1^; mobile phase: acetonitrile and 0.1% phosphoric acid water (44:56, v/v) [30].

### Study on the stability of liposomes

After the preparation of TPP-SS-ATS-LS, they were sealed and stored at 4 ℃. TPP-SS-ATS-LS were taken at 0, 7, 14, 28 and 42 days, respectively to determine the particle size, entrapment efficiency, zeta potential and PDI. The stability of liposomes was evaluated. The determination methods of relevant parameters were shown in “Characterization of TPP-SS-ATS-LS”.

### Molecular dynamics simulation

The molecular dynamics simulation was carried out by Gromacs2020.02 software, and the simulation was accelerated by GPU (GeForce RTX 3080). The charmm36 force field was used to generate topological files of phospholipids, cholesterol and TPP-SS-ATS molecules [31]. The gmx insert command was executed to add 128 PLPC phospholipid molecules, 13 TPP molecules and 2000 SPC water molecules to a 10 nm cube box [32]. The conjugate gradient method was used to optimize 5000 steps, and the cutoff value of each step was set to 0.1 nm. Before the simulation, the system was pre-balanced at 200 ps, and then the simulation was performed at 1000 ns.

### Cytotoxicity analysis

The cytotoxicity of different conjugates and ATS on 4T1, MCF-7 and MDA-MB-231 cells were determined using CCK-8 method. The cells were seeded in 96-well culture plates at a density of 3000–5000 cells/well. Then the cells were treated with conjugates and free ATS at concentrations ranging from 2.5 to 160 μM at 37 ℃ overnight when 60–70% confluence was reached. After co-incubation, CCK-8 was added into the drug solution and co-incubated with cells for another 2 h. The absorbance at 450 nm was recorded using EnVision microplate reader (PerkinElmer, USA). The survival rate was calculated by the following formula:

$$Survival\;rate\;(\% ) = \frac{ODtest}{{ODcontrol}} \times 100\% .$$

### Monoclonal formation experiment

MDA-MB-231 cells were incubated in six-well plates (1000/well) overnight and then treated with various concentrations (0, 0.1, 0.3, 1, 3, 10 μM) of TPP-SS-ATS-LS for 24 h. Then replaced the medium with a normal fresh complete medium, and the solution was changed every 3 days. The formation of clones was observed under an optical microscope. The culture was terminated after 7 days, when visible clones appeared in the plates. The supernatant was discarded, washed with PBS and fixed with methanol. The fixing solution was discarded, 1 mL of 1% crystal violet-methanol staining solution was added to each hole for 30 min, and then washed away slowly the stain with flowing water and took photos after the well dried.

### In vitro tumor targeting evaluation

MDA-MB-231 cells were seeded into 96-well culture plates at a density of 2000 cells/well and incubated for 12 h. Then the mitochondria were stained with Mito Tracker Red CMXros and the nucleuses were stained with Hoechst 33342 according to the instructions. After washing with PBS, cells were cultured with 20 μM FITC-TPP-SS-ATS-LS in complete medium. The cells were monitored using confocal scanning imaging for 48 consecutive hours with PE Perkin Elmer Operetta CLS™ (PerkinElmer, USA). The intake process of the TPP-SS-ATS-LS was recorded hourly.

### Ultrastructure observation under transmission electron microscopy

A total of 2 × 10^6^ cells were inoculated into 10 cm culture dish and administered with 20 μM TPP-SS-ATS-LS or not. After 24 h, all cells were washed, scraped off with 2.5% glutaraldehyde, and centrifuged (1000 rpm for 5 min). Precipitated cell clumps were fixed with 2.5% glutaraldehyde at room temperature for 2 h. Then transfer to 4 ℃, washed with PBS, mounted with 1% osmic acid for 2 h, dehydrated in ethanol and acetone, and Epon812 embedded. After polymerization, the samples were sliced using an ultrathin slicing machine (Leica UC7), counterstained with 2% oil and citric lead acetate and observed under TEM (Hitachi, HT7800).

### In vivo targeting evaluation tumor implantation

The 4T1 tumor-bearing mice model was established based on previously reported method [33]. Briefly, axillary tumor containing 4T1 cells was extracted and diluted in normal saline with 1 × 10^7^ cells/ml. The mice were subcutaneously injected with 0.2 ml of cell suspension for breast tumor implantation. DiR-TPP-SS-ATS-LS were administered intraperitoneally when the tumor length reached 0.8–1 cm. The tumor-bearing mice were anesthesia with isoflurane, an *IVIS Lumina III Imaging System* (PerkinElmer, USA) was applied for determining the fluorescence from DiR-TPP-SS-ATS-LS at intervals of 4, 8, 12, 24, 36, 48, 60, 72, 96, 120, 144 and 168 h post-injection.

### In vivo anti-tumor efficacy

The 4T1 tumor-bearing mice model was established as above and the mice were randomly divided into six groups. The control group mice were administered saline. The other groups were administered free ATS, and equivalent TPP-SS-ATS-LS with 15 mg/kg or 30 mg/kg intraperitoneally injected once every 2 days. As a positive control, 30 mg/kg gemcitabine was administered intraperitoneally every 4 days. Body weights were recorded every 3 days. After taking blood, the mice were sacrificed by euthanasia on day 22. The body weight and blood hematological and biochemical indicators of mice was recorded to evaluate the systematic toxicity of different groups. The TGI (tumor growth inhibition ratio) was calculated according to the following formula:

$$TGI\,(\% ) = \frac{C - T}{C} \times 100\% ,$$

where “T” represented the average weight of treated groups, and “C” represented the average weight of control group.

### Bulk RNA sequencing and data analysis

The 4T1 tumor-bearing mice model was established as above and the mice were randomly divided into model, 30 mg/kg ATS and TPP-SS-ATS-LS groups. RNA was isolated from the tumor samples using the Qiagen RNeasy^Ⓡ^ Mini Kit according to the manufacturer's recommendations. The isolated RNA of each sample was enriched for poly-A templates, and further used for mRNA sequencing on the Illumina Novaseq 6000 sequencer (Illumina, San Diego, CA, USA) with paired-end 150 bp (PE150) reads. Raw sequencing data were submitted to quality control using fastp as previously mentioned [34], then reads were aligned to the mouse reference genome mm10 using STAR [35] (version 2.2.1). Reads quantification was performed using the featureCounts [36] (version 1.5.0). Next, DEGs analysis was performed using the limma. P values were generated from the empirical Bayes test model and were adjusted using Benjamini–Hochberg (BH) method. The genes with absolute fold change ≥ 2 and adjusted P value (FDR) < 0.05 were considered to be significant DEGs in our research. Gene Ontology (GO) analysis was performed using the clusterProfiler R package (version 3.18.1) [37], according to the up and down-regulated protein identified by DEGs analysis. P values were generated from the Hypergeometric test model and adjusted using BH. The biological process (BP) category was selected to represent the functional profiles, and visualized based on the count of proteins enriched and the adjusted P value (Q value) < 0.05.

### Hemolytic assay

The red blood cells (RBC) were obtained from BALB/c mice and centrifuged at 2400 rpm for 5 min then discarded the leukocytes and supernatant. 2% RBC in PBS was incubated with TPP-SS-ATS-LS for 1 h at 37 ℃. The positive and negative control was RBC incubated with 1% Triton X-100 and with PBS, respectively. The sample was centrifuged at 5000 rpm for 5 min. Then the supernatant was analyzed at 540 nm by the EnVision microplate reader. The Hemolytic rate was calculated by the following formula:

$${\text{Hemolytic rate}}(\% ) = \frac{{OD_{sample} - OD_{negative} }}{{OD_{positive} - OD_{negative} }}\, \times 100\% .$$

### Histological and immunofluorescence evaluation studies

After the end of the anti-tumor efficacy experiment, the mice were sacrificed by euthanasia and the hearts, livers, kidneys and brains of the mice were taken out. The organs were fixed with 10% formalin, embedded in paraffin, sectioned, stained with H&E (Hematoxylin–eosin) and the tumor tissues were detected with immunofluorescence evaluation. The organs were observed and photographed with BX51 optics microscope (Olympus, Japan). Mice tumor paraffin tissues were incubated in antigen retrieval for 10 min at room temperature. The slices with tumor embedded were blocked with 5% BSA 1 h at room temperature, and incubated with primary antibodies against LC3 or PHB2 at 4 ℃ overnight. Then the slices were washed three times with TBST and incubated for 2 h at room temperature with secondary fluorescence antibodies (goat anti-rabbit, 1:500; goat anti-mouse, 1:500, Abcam) avoiding from light. After 10 min of Hoechst staining, the slices were sealed and then photographed with TCS SP8 CARS laser scanning confocal microscope (Leica, Germany).

### Mitochondrial respiration function measurement

The XF96 extracellular flux analyzer (Seahorse Bioscience, Billerica, USA) was used to detect real-time changes in basal and maximal respiration (OCR) for the assessment of mitochondrial function. All the agents were supplied by Agilent Seahorse XF (USA). Briefly, the culture plate was seeded with 4T1 (2 × 10^4^ cells per well), MDA-MB-231 (2 × 10^4^ cells per well) and MCF-7 (4 × 10^4^ cells per well) and cultured overnight. Cells were treated with 20 μM of ATS, TPP-SS-ATS or TPP-SS-ATS-LS for 24 h prior to the measurement. Then, the cells were washed 3 times. 1 mM pyruvate (103578-100) and 2 mM glutamine solution (103579-100) was added in XF RPMI (103576-100) and DMEM (103575-100) Base Medium for washing and culture. For OCR analysis (Seahorse XF Cell Mito Stress Test Kit, Cat No. 103015-100, USA), final concentration of 2 µM Oligomycin, 1 µM FCCP and 0.5 µM Rotenone/ Antimycin A were added. All measurements were performed following manufacturer’s instructions and normalized with the number of cells counted with Hoechst33342 in each well at the end of the seahorse experiments.

### Western blot analysis

After TPP-SS-ATS-LS treatments, MDA-MB-231 cells lysate were obtained with RIPA lysate and protease inhibitor. The supernatants were collected and centrifuged to discard cell debris. Protein concentration was determined using the BCA protein Assay Kit, and the denatured sample was separated with 10% or 15% SDS-PAGE gel, respectively. The samples were transferred onto PVDF membranes, and then the membranes were blocked with 5% bovine serum albumin (BSA) for 1 h at room temperature. Membranes were incubated at 4 ℃ overnight with primary antibody in 3% BSA. After washing with TBST, the membranes were incubated with horseradish peroxidase-conjugated secondary antibodies for 2 h at room temperature and washed three times with TBST again. The bands were visualized using enzyme-linked chemiluminescence in the Enhanced Chemiluminescence Plus detection system (Azure C400, USA). The density of each band was quantified using Image J software. The data were expressed as ratios and were normalized to the amount of β-actin or GAPDH.

### Statistical analysis

Data were presented as the mean ± standard deviation of at least three repeated samples. Differences between groups were analyzed using Graphpad Prism and One-way ANOVA followed by Dunnett’s tests was performed. The value of *P* < 0.05 was considered statistically significant.

## Results and discussion

### Chemical structure confirmation of the chemical intermediate (SS-ATS) and the GSH-sensitive artesunate smart conjugate (TPP-SS-ATS)

#### Chemical structure confirmation of SS-ATS

SS-ATS (Fig. 1A, Additional file 1: Fig. S1) was obtained as an oily substance. Its molecular formula was C_23_H_36_O_9_S_2_ with MS at *m/z*: 559.1 [M + K]^+^. The ^1^H NMR spectra in CDCl_3_ revealed that the characteristic protons signals of artesunate *δ*_H_ 2.37 (1H, brt, *J* = 13.5 Hz, H-4α), 2.03 (1H, brd, *J *= 14.5, H-4β), 1.43–1.52 (1H, m, H-5α), 1.88–1.94 (1H, overlapped, H-5β), 1.27–1.30 (1H, m, H-5a), 1.34–1.36 (1H, m, H-6), 0.96–1.05 (1H, m, H-7α), 1.72 (1H, overlapped, H-7β), 1.36–1.38 (1H, overlapped, H-8α), 1.78 (1H, brd, *J* = 13.3 Hz, H-8β), 1.62 (1H, br d, J = 13.9 Hz, H-8a), 2.53–2.59 (1H, m, H-9), 5.78 (1H, d, J = 9.8 Hz, H-10), 5.44 (1H, s, H-12), 1.43 (3H, s, H-14), 0.96 (3H, d, *J* = 5.0 Hz, H-15), 0.86 (3H, d, *J* = 6.6 Hz, H-16). The above ^1^H NMR and MS spectra (Additional file 1: Figs. S3, S5) confirmed that SS-ATS was successfully synthesized.

#### Chemical structure confirmation of TPP-SS-ATS

TPP-SS-ATS (Fig. 1A; Additional file 1: Fig. S2) was obtained as white amorphous solid. Its molecular formula was C_45_H_56_BrO_10_PS_2_ with MS at *m/z*: 851.4 [M-Br]^+^. The ^1^H NMR with HSQC spectrum in CDCl_3_ revealed that the protons signals of artesunate structure fragment at *δ*_H_ 2.37 (1H, td, *J* = 14.0, 3.8 Hz, H-4α), 2.03 (1H, dt, *J* = 14.5, 3.8 Hz, H-4β), 1.43–1.52 (1H, m, H-5α), 1.87–1.92 (1H, m, H-5β), 1.27–1.30 (1H, m, H-5a), 1.33–1.34 (1H, m, H-6), 0.96–1.07 (1H, m, H-7α), 1.72 (1H, dd, *J* = 13.4, 3.0 Hz, H-7β), 1.37 (1H, dd, *J* = 13.5, 3.3 Hz, H-8α), 1.78 (1H, dd, *J* = 13.6, 3.6 Hz, H-8β), 1.62 (1H, dt, *J* = 13.7, 4.4 Hz, H-8a), 2.54–2.56 (1H, m, H-9), 5.78 (1H, d, *J* = 9.8 Hz, H-10), 5.31 (1H, s, H-12), 1.41 (3H, s, H-14), 0.96 (3H, d, *J* = 6.1 Hz, H-15), 0.85 (3H, d, *J* = 7.1 Hz, H-16); the protons signals of 2,2-dithiodiethanol fragment were at *δ*_H_ 4.32 (4H, dd, *J* = 15.9, 6.6 Hz, H-2′ and 7′) and 2.90–2.91 (4H, m, H-3′ and 6′); the protons signals of (4-carboxybutyl)triphenylphosphonium bromide fragment were at *δ*_H_ 2.92–2.94 (2H, m, H-b), 1.93–1.99 (2H, m, H-c), 4.00–4.05 (2H, m, H–d) and 7.70–7.90 (15H, m, H-TPP aromatic protons) (Fig. 7). The characteristic signals of the ^13^C NMR spectra were as follows: *δ*_C_ 171.1 (C-17, ester carbonyl of ATS), 171.9 (C-20, ester carbonyl of ATS), 62.5 and 62.6 (C-2′ or C-7′), 36.9 and 37.0 (C-3′ or C-6′), 172.9 (C-a, ester carbonyl of TPP), 117.9–135.1 (C-f, TPP aromatic carbons). The location of (4-carboxybutyl)triphenylphosphonium bromide fragment linked to C-20 of artesunate fragment was verified by the HMBC correlation between *δ*_H_ 4.32 (H-2′) and *δ*_C_ 171.9 (C-20); the location of the TPP fragment linked to C-7′ of 2,2-dithiodiethanol fragment was verified by the HMBC correlations between *δ*_H_ 4.32 (H-7′) and *δ*_C_ 172.9 (C-a), and *δ*_H_ 1.93–1.99 (H-c) and *δ*_C_ 172.9 (C-a) (Fig. 1). In general, all the above spectra of 1D, 2D NMR and MS spectra (Additional file 1: Figs. S4, S6, S7–S9) confirmed that TPP-SS-ATS was successfully synthesized, and the purity of this conjugate determined by HPLC was 97.25% (Additional file 1: Fig. S10, Table S1, the ultraviolet spectrogram of TPP-SS-ATS was showed in Additional file 1: Fig. S11).

### Characteristics of liposomes

**Fig. 2:**
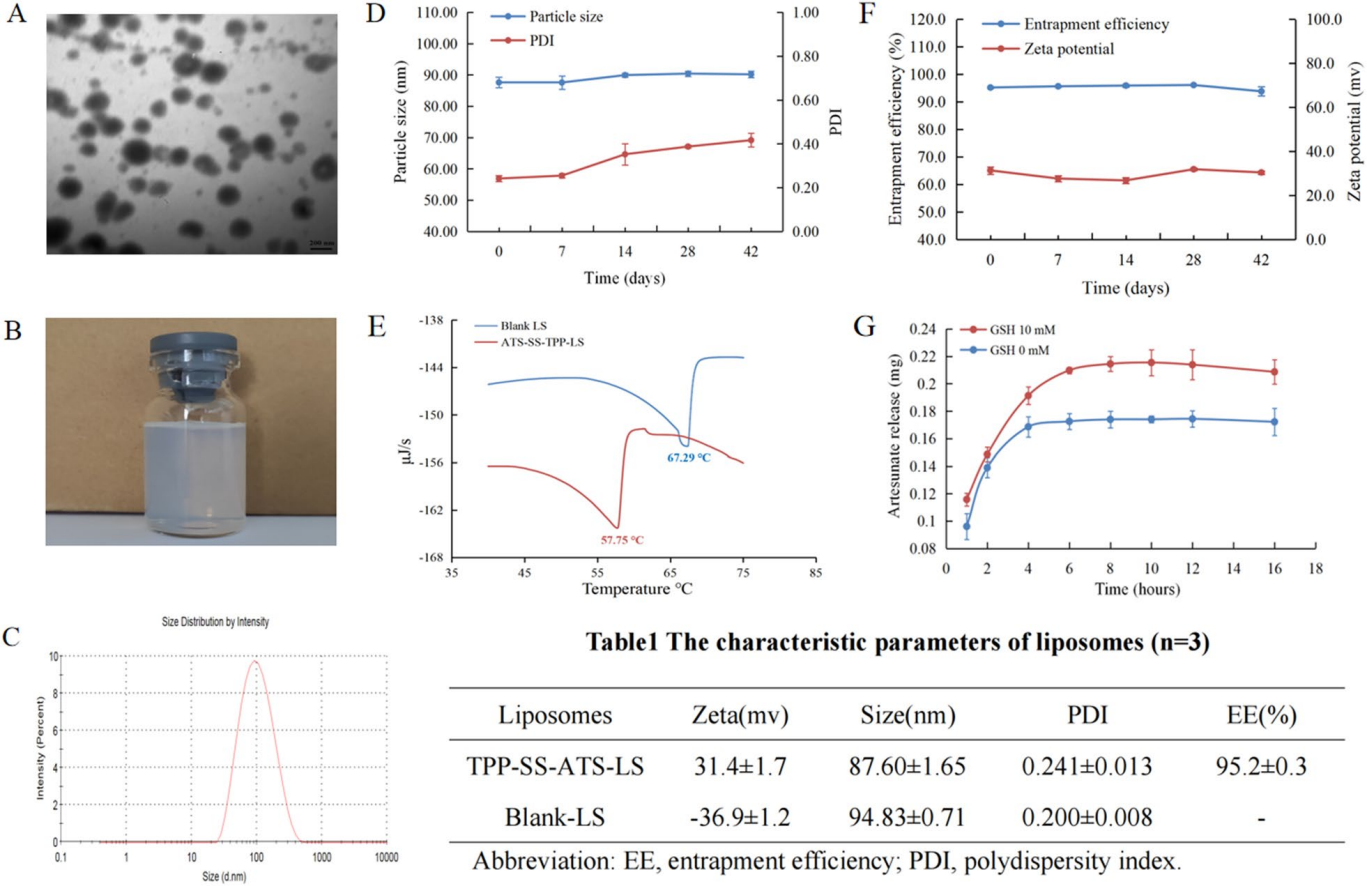
**A** Transmission electron microscopy image of TPP-SS-ATS-LS. **B** The appearance of TPP-SS-ATS-LS. **C** The particle size image of TPP-SS-ATS-LS. **D**, **F** The results of the stability studies of TPP-SS-ATS-LS at 4 ℃ (n = 3). **E** The phase transition temperatures of liposomes. **G** Release of ATS from TPP-SS-ATS-LS in GSH and non-GSH environments (n = 3)

The particle size, zeta potential, PDI, entrapment efficiency (EE), drug loading (DL) and phase-transition temperature of TPP-SS-ATS-LS were 87.60 ± 1.65 nm, 31.4 ± 1.7 mV, 0.241 ± 0.013, 95.2 ± 0.3%, 0.93 ± 0.00% and 57.75 ℃, respectively. The particle size, zeta potential, PDI and phase-transition temperature of Blank-LS were 94.83 ± 0.71 nm, − 36.9 ± 1.2 mV, 0.200 ± 0.008 and 67.29 ℃, respectively. The appearance of TPP-SS-ATS-LS was as a translucent liquid, showing light blue opalescence and the TEM result showed that the morphology of TPP-SS-ATS-LS was regular spherical. The results are shown in Fig. 2A–C, E.

TPP-SS-ATS is a positively charged conjugate molecule. After the molecule was encapsulated in the liposome, its membrane potential changed from − 36.9 ± 1.2 mV to 31.4 ± 1.7 mV. Moreover, the phase transition temperature of liposomes decreased from 67.29 ℃ to 57.75 ℃. These results could indirectly prove that TPP-SS-ATS was loaded in the lipid membrane structure of liposomes and affected its physical parameters. This structural feature of TPP-SS-ATS-LS was conducive for recognition by mitochondria under the traction of triphenylphosphine structural fragment (TPP).

### Molecular dynamics simulation of liposome structure formation

In this study, the trajectory images of 0, 100, 200, 450, 600, 750, 900 and 1000 ns in the molecular dynamics simulation trajectory file were sequentially cut out (Additional file 1: Fig. S12). The images directly showed that liposomes loaded with TPP-SS-ATS began to form a bilayer membrane structure at 200 ns, and form a typical bilayer structure after 900 ns. In this process, the system changed from disorder to order. Finally, the hydrophobic tail of the phospholipid gathered in the center of the membrane structure, the hydrophilic head fragments were located on the outside, and molecules of TPP-SS-ATS were located at the edge of the membrane structure. The TPP-SS-ATS located on the outer side has TPP (triphenylphosphine structural fragment) in its structure, which is conducive to the capture of TPP-SS-ATS-LS by mitochondria. This structural feature could improve the mitochondrial targeted delivery ability of the liposomes.

### Liposome stability

The appearance of TPP-SS-ATS-LS remained as a translucent liquid, showing light blue opalescence after being placed at 4 ℃ for 42 days. On the 0, 7, 14, 28 and 42 days after the preparation of liposomes, the RSD% of particle size and EE were 1.60% and 0.96%, respectively. During the stability study, the Zeta potential of TPP-SS-ATS-LS was always greater than 26 mV. These results showed that TPP-SS-ATS-LS had good stability at 4 ℃. The relevant data of the stability studies are shown in Fig. 2D, F.

### *Drug release *in vitro

The in vitro release characteristics of artesunate from TPP-SS-ATS-LS in the absence and presence of GSH (Glutathione) are shown in Fig. 2G. Although the release of artesunate in the GSH group was greater than that in the non-GSH group, GSH had no significant effect on the release of artesunate in the first two hours after the start of the release experiment. This situation changed with the progress of the experiment. GSH had a significant effect on the release of artesunate from 4 h. At the 8th hour sampling point, the artesunate release of TPP-SS-ATS-LS was increased by about 30% with the help of GSH in the dissolution media. The above results showed that GSH could promote the breaking of disulfide bonds in the molecular structure of the prodrug (TPP-SS-ATS-LS) and accelerate the release of artesunate, so as to realize the intelligent release of artesunate in the high GSH environment of tumor cells.

### The proliferation and targeting analysis

**Fig. 3:**
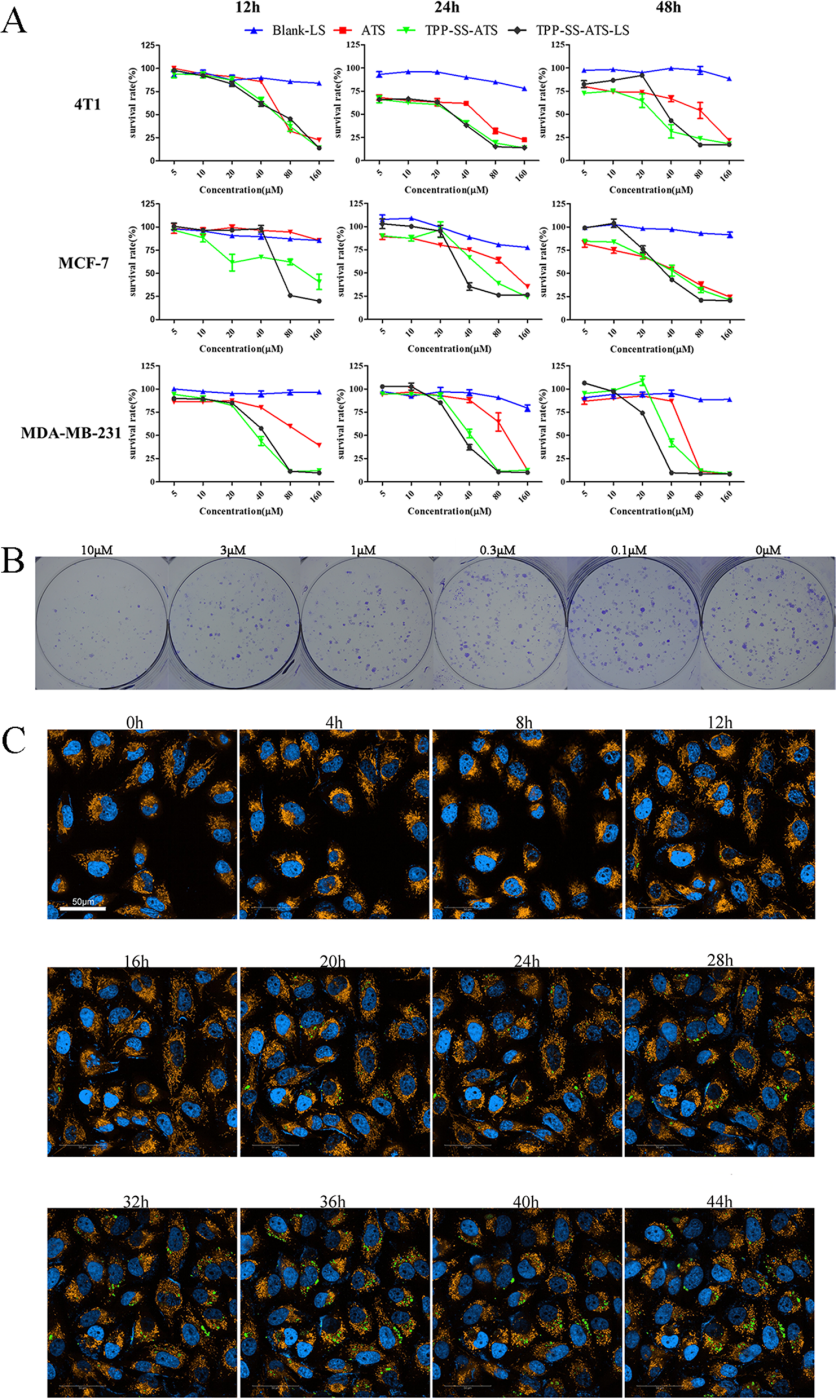
TPP-SS-ATS-LS on proliferation of breast cells and targeting ability. **A** The cytotoxicity of TPP-SS-ATS-LS Dose response curves of the cytotoxic effects of TPP-SS-ATS-LS in 3 breast tumor cell lines. Data represent the means of triplicate samples ± standard error (SD, n = 3). **B** Effect of TPP-SS-ATS-LS on the ability of cells monoclonal forming. **C** Confocal images of MDA-MB-231 cells incubated with TPP-SS-ATS-LS for continuous period. TPP-SS-ATS-LS entry into mitochondria begins at 6 h (green fluorescent spot), and gradually accumulates in the mitochondria during the detected 48 h. (scale bar: 50 μm)

Cell viability assay showed that TPP-SS-ATS-LS and TPP-SS-ATS could both obviously inhibit the growth of different breast cancer cells in a dose-dependent manner. TPP-SS-ATS-LS showed a more pronounced antitumor effect than ATS. During the detected 72 h, the IC50 value of TPP-SS-ATS-LS ranged from 17.69 μM (4T1 cells, 48 h) to 67.48 μM (MCF-7 cells, 12 h), while the value of ATS ranged from 33.50 μM (4T1 cells, 48 h) to 797.5 μM (MCF-7 cells, 12 h). The cytotoxicity of TPP-SS-ATS-LS was obviously higher than that of ATS at the same dosage, but there is no significant difference between TPP-SS-ATS-LS and TPP-SS-ATS (Fig. 3A).

Cloning forming experiment is another effective method to determine the proliferative capacity of a single cell. To test the effect of TPP-SS-ATS-LS on the ability of breast cancer cells to form monoclonal cells, we treated MDA-MB-231 cells with different concentrations of TPP-SS-ATS-LS for 24 h. The results (Fig. 3B) showed that the number of clones in the administration group was obviously reduced compared with the control group, showing the concentration-dependent inhibition of the MDA-MB-231 cell monoclonal forming ability by TPP-SS-ATS-LS. The study demonstrated that TPP-SS-ATS-LS can not only inhibit the survival ability but also suppress monoclonal forming of the breast cancer cells.

The breast cell uptake behavior of TPP-SS-ATS-LS was subsequently monitored using a micro confocal high-content imaging system. During the detected 48 h, TPP-SS-ATS-LS entry into mitochondria gradually accumulated. At the sixth hour, TPP-SS-ATS-LS was first perceived in the mitochondria (Fig. 3C). The smart liposomes were thus able to rapidly enter tumor cells and exhibit stronger cytotoxic effects.

### In vivo* anti-tumor studies*

**Fig. 4:**
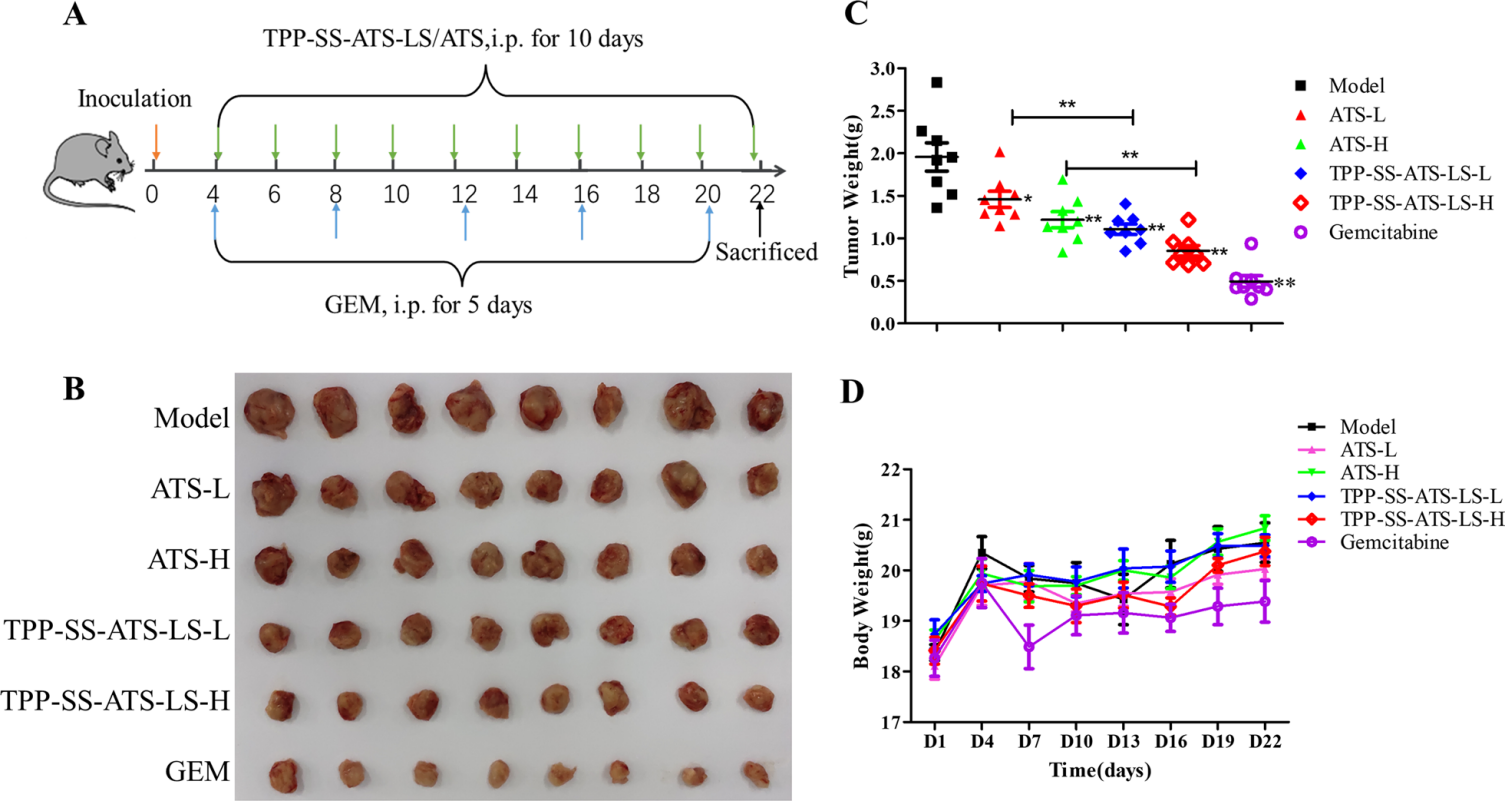
Anti-tumor effect of liposomes in mice. **A** Schematic illustration of the administration. The red arrow indicates the tumor cell inoculating. The green and blue arrows indicate the administration. And the black arrow indicates the experiment ending point. **B** Tumor volume changes in each group during the experiment (mean ± SD, n = 8). L group stands for 15 mg/kg and H group stands for 30 mg/kg. **C** The Effect of liposomes on mouse 4T1 tumor weight (mean ± SD, n = 8). *P < 0.05, **P < 0.01. **D** Body weight changes in each group during the experiment (mean ± SD, n = 8)

Xenografts tumors could be felt in breast tissue 4 days following the injection. In vivo experiments showed that tumor weights were obviously reduced after administration. Unlike gemcitabine, both ATS and TPP-SS-ATS-LS had no effect on body weight of tumor bearing mice. Compared with the model group, the inhibition effect of 30 mg/kg ATS was 37.7%, while the tumor growth inhibition ratio (TGI) of equivalent TPP-SS-ATS-LS was nearly 56.4%, which was significantly better (Fig. 4). As speculated, the antitumor effect of TPP-SS-ATS-LS was significantly enhanced compared with that of equivalent ATS.

In addition, there was no significant diversity of body weight in all experimental groups (Fig. 4D). To further study the therapeutic effect, the tumor and other normal tissues were examined by H&E staining after being treated for 22 days.

To evaluate whether TPP-SS-ATS-LS caused hemolysis, a hemolytic assay was performed. It was found that the hemolysis rate was less than 5% at high doses in pharmacological experiments. It’s showed that the TPP-SS-ATS-LS were safe. The results are shown in Additional file 1: Fig. S13.

### Histological evaluation studies

**Fig. 5:**
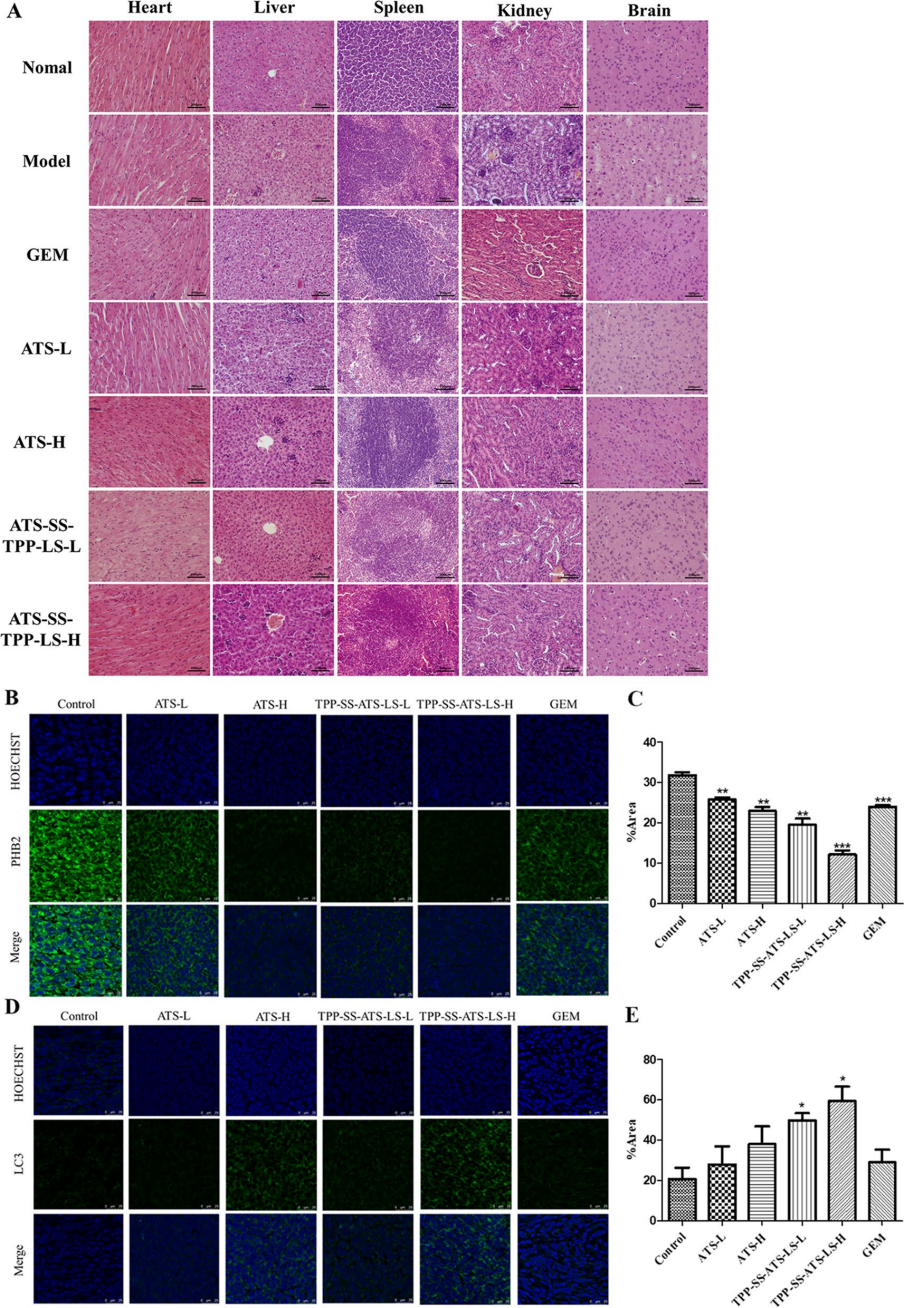
In vivo pathology and cancer inhibition evaluation. **A** Representative photomicrographs of HE stained (× 400) of organs. **B**, **C** PHB2 and **D**, **E** LC3 stained (× 400). The mean value was calculated by the t test (mean ± SD, n = 3). Compared with Control group, *P < 0.05, **P < 0.01

After administrating with TPP-SS-ATS-LS, the organs (heart, liver, spleen, kidney and brain) were examined pathologically. The examination revealed that the heart, liver, spleen, kidney, and brain tissues of mice treated with the TPP-SS-ATS-LS were similar to those of mice in the normal saline treatment group and no pathological changes were observed (Fig. 5A). The results are as follows: (1) Heart: The morphology and structure of cardiomyocytes in the blank group, model group and each administration group are normal, the myocardial fiber cells are arranged orderly, the transverse lines are clear, the cell membrane is complete and the staining is uniform. (2) Liver: The vacuolar degeneration of hepatocytes in the GEM group is more obvious; the pathological changes in the liver of the other administration groups are similar to those of the model group, which means that TPP-SS-ATS-LS and ATS will not aggravate the liver damage of the model mice. (3) Spleen: The junction of spleen cortex and medulla was obvious in blank group, model group and each administration group, and there is a dense distribution of lymphocytes in the white pulp and red pulp. (4) Kidney: The renal tubular epithelial cells of the mice in the blank group, model group and each administration group are arranged neatly, the renal capsule cavity is normal, and the glomerular structure is clear and uniform in size. (5) Brain: The neuronal cells and glial cells in the brain tissue of the mice in the blank group, the model group and each administration group are tightly arranged without cell swelling. These results show that there was no obvious toxicity of TPP-SS-ATS-LS to various organs of mice.

PHB2, being located in the inner mitochondrial membrane, is an important member of the prohibitin (PHB) family and functions as a mitophagy receptor. PHB2 binds to LC3 (short for human microtubule-associated protein light chain 3) through LC3 interaction domain (LIR) to induce the degradation of damage mitochondria [38]. LC3-II is a key mitochondrial autophagy marker that participates in mitophagy, and it is well known that autophagy plays an important role in the development of cancer. PHB2 also play an important role in the proliferation and survival of cancer cells through mitochondrial apoptosis [39–41], and down regulating the expression of PHB can significantly reduce the cell division rate [42, 43]. Meanwhile, increasing evidence indicates that the PHB2-mediated signaling pathway is essential for the inhibition of cancer cell proliferation and migration [44–46].

As shown in Fig. 5B, TPP-SS-ATS-LS caused the largest region of cell death in the tumor tissue, while there was no obvious damage in the normal tissues. In accord with the results of immunofluorescence detection, TPP-SS-ATS-LS significantly influenced the expression of PHB2 (Fig. 5B, C) and LC3-II (Fig. 5D, E) in tumor tissues in a dose-dependent manner (*P* < 0.05). Meanwhile, the anticancer efficacy of ATS was obviously weaker than equivalent conjugates. Overall, these results demonstrated that the TPP-SS-ATS-LS mediated the inhibition of tumor cell proliferation though induction of autophagy.

### In vivo* potential side effects evaluation*

**Fig. 6:**
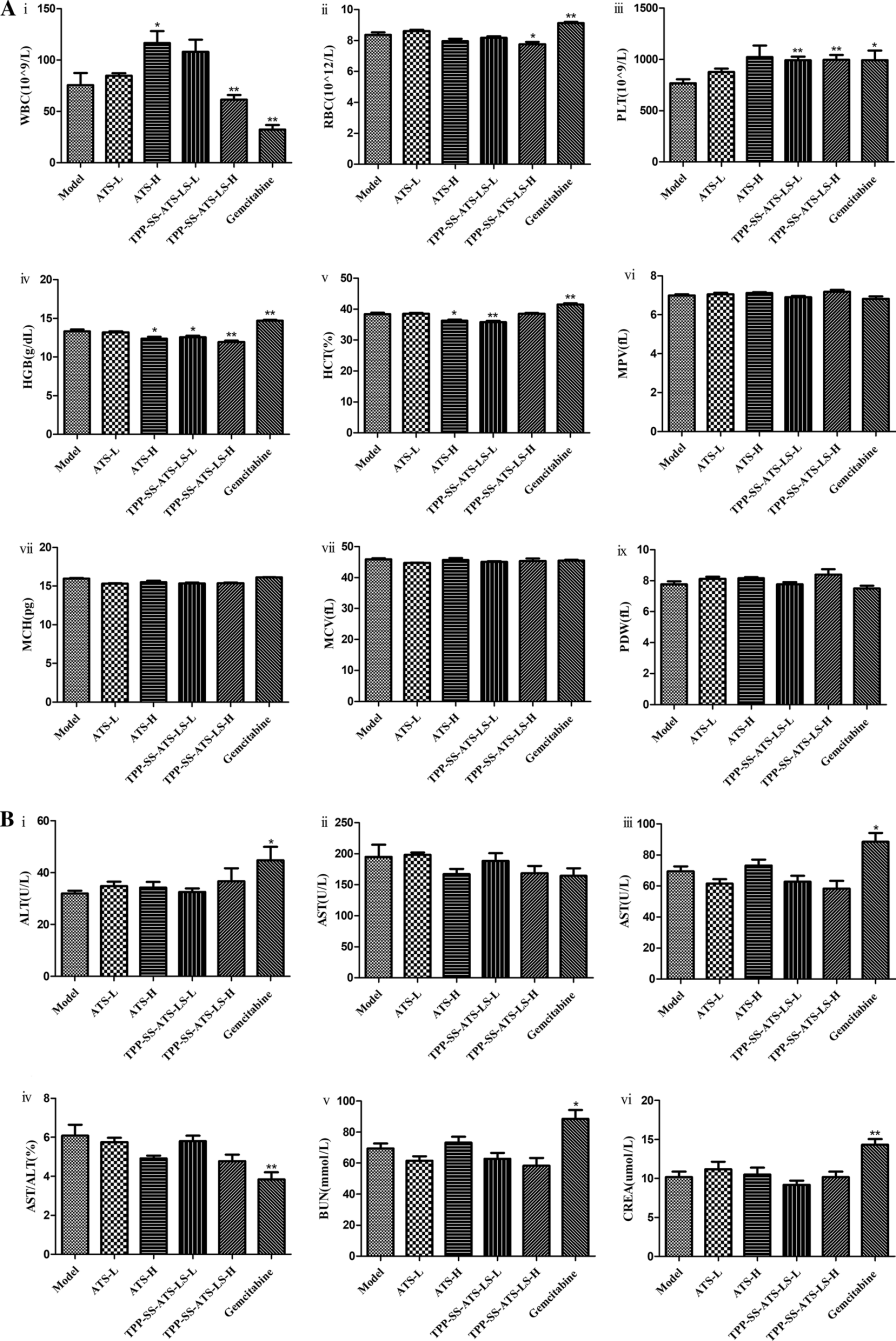
In vivo side effects evaluation. **A** Hematological data of the mice intraperitoneal injected with different samples at the 22ed day post-injection. The terms are noted as followed: (i) white blood cells (WBC), (ii) red blood cells (RBC), (iii) platelets (PLT), (iv) haemoglobin (HGB), (v) haematocrit (HCT), (vi) mean platelet volume (MPV), (vii) mean corpuscular haemoglobin (MCH), (viii) mean corpuscular volume (MCV), and (ix) platelet distribution width (PDW). **B** Blood biochemical analysis at the 22ed day post-injection. The terms are following: (i) alanine transaminase (ALT), (ii)aspartate transaminase (AST), (iii)alkaline phosphatase (ALP), (iv)AST/ALT, (v)blood urea nitrogen (BUN) and (vi)creatinine (CR). Compared with Control group, *P < 0.05, **P < 0.01

For further study of the in vivo toxicology and potential side effects, hematological indicators were investigated systematically. The standard hematological markers including the white blood cells (WBC), red blood cells (RBC), platelets (PLT), haemoglobin (HGB), haematocrit (HCT), mean platelet volume (MPV), mean corpuscular haemoglobin (MCH), mean corpuscular volume (MCV), and platelet distribution width (PDW) were analyzed (Fig. 6A). Compared with the model group, high dose of TPP-SS-ATS-LS and gemcitabine obviously affected WBC, RBC, PLT and HGB level (*P* < 0.05), all the other parameters in the treated groups appeared to be normal and there were no statistically significant differences between both groups (*P* > 0.05). These results indicated that these treatments did not cause obvious injury and inflammation in the TPP-SS-ATS-LS treated mice. Blood biochemical analysis were carried out and the parameters about the functions of the liver and kidney of mice including alanine transaminase (ALT), aspartate transaminase (AST), alkaline phosphatase (ALP), blood urea nitrogen (BUN), and creatinine (CR) were examined (Fig. 6B). Unlike gemcitabine, no meaningful difference was detected between the treated groups and the model control group. Hence, the TPP-SS-ATS-LS treatment did not affect the blood chemistry of mice. These blood biochemical results demonstrated that TPP-SS-ATS-LS treatment induced no obvious hepatic and kidney toxicity in mice.

### In vivo tumor targeting evaluation

**Fig. 7:**
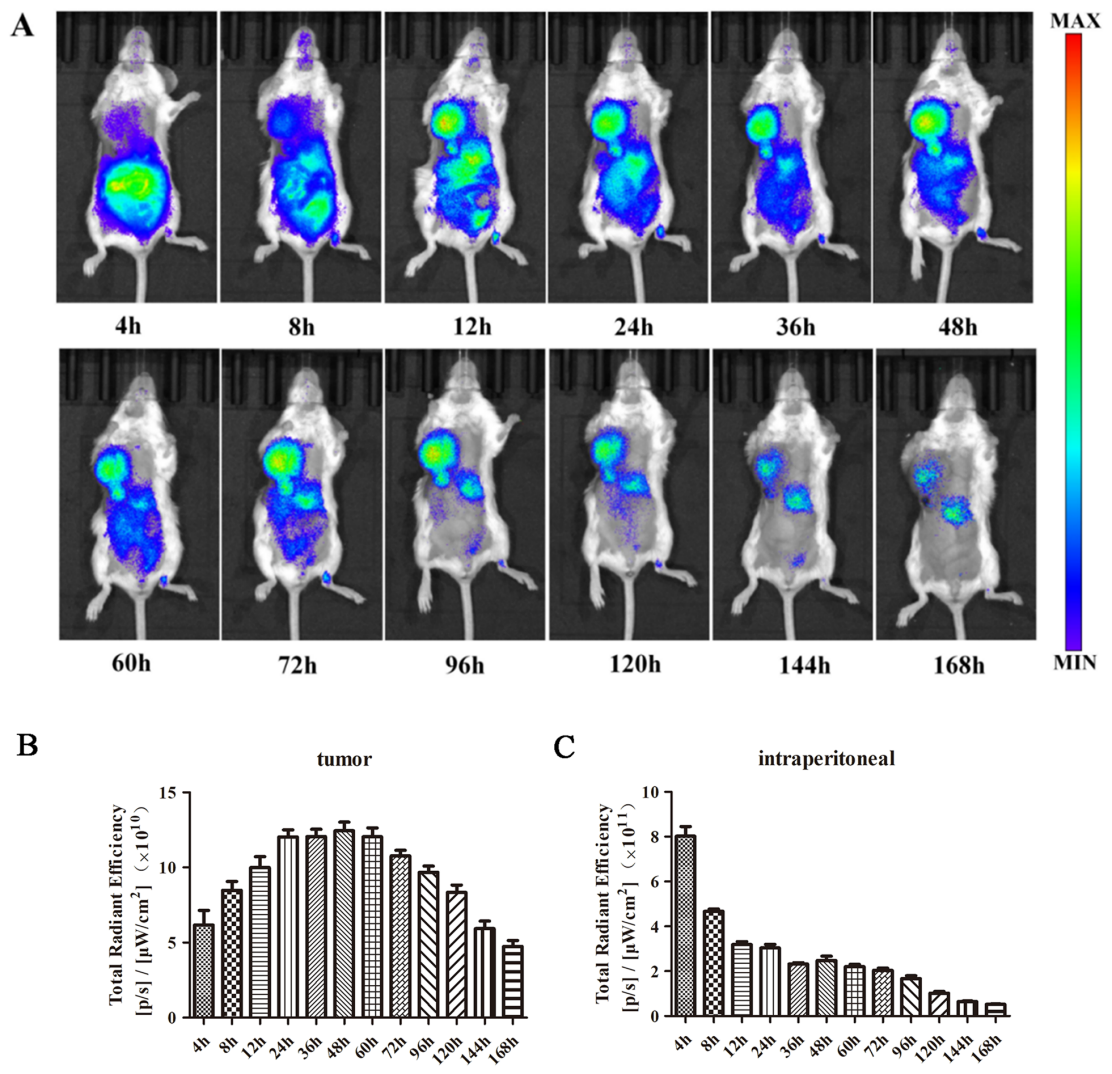
In vivo targeting evaluation of TPP-SS-ATS-LS (Ex/Em: 670/770 nm). **A** In vivo fluorescence imaging of 4T1 tumor-bearing Balb/c mice after intraperitoneal injection with DiR-TPP-SS-ATS-LS. Quantitative analysis for the fluorescence intensity of tumor sites (**B**) and inject sites (**C**) in A. The mean value was calculated by the t test (mean ± SD, n = 6)

Further, we monitored the tumor targeting property of TPP-SS-ATS-LS in 4T1 orthotopic implanted tumor-bearing mice. The results of in vivo imaging studies demonstrate the targeting of TPP-SS-ATS-LS as shown in Fig. 7. In the study, TPP-SS-ATS-LS was injected into the model animals by intraperitoneal injection, and the fluorescence intensity at the tumor site gradually increased over time (Fig. 7A, B), while the fluorescence intensity at the administration site (abdominal cavity) gradually weakened (Fig. 7A, C). The fluorescence signal intensity at the tumor site reached a highest level at 24–60 h after administration, and then gradually decreased. The possible process of targeting tumor tissue after intraperitoneal injection of TPP-SS-ATS-LS was as follows [47]: after administration, the liposomes entered the systemic circulation through the capillaries and lymphatic vessels in the peritoneum, and then the liposomes were gradually captured by the tumor tissue to achieve the effect of tumor-targeted delivery.

### Bulk RNA sequencing data analysis

**Fig. 8:**
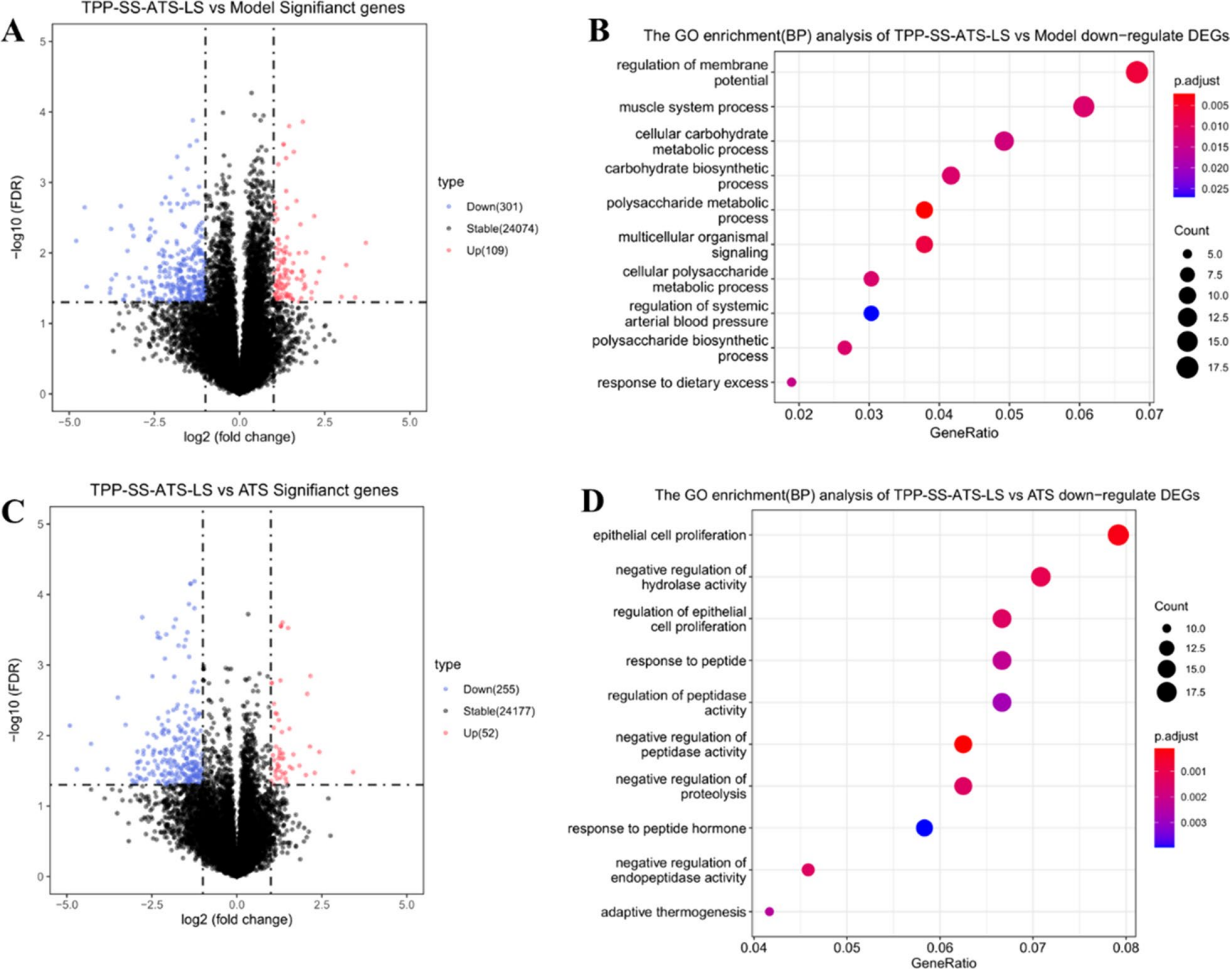
DEGs and BP analysis of TPP-SS-ATS-LS/Model and TPP-SS-ATS-LS/ATS comparison. **A** Volcano plot of differently expressed genes in TPP-SS-ATS-LS/Model comparison. **B** GO enrichment analysis of down-regulated DEGs in TPP-SS-ATS-LS/Model comparison showing the top 10 categories. **C** Volcano plot of differently expressed proteins in TPP-SS-ATS-LS/ATS comparison. **D** GO enrichment analyses of down-regulated DEGs in TPP-SS-ATS-LS/ATS comparison showing the top 10 categories. *GO* Gene Ontology, *DEGs* differentially expressed genes

To identify the altered gene expression and enriched biological pathways between TPP-SS-ATS-LS and the model group, we performed differentially expressed genes (DEGs) analysis and revealed a total of 410 (109 up, 301 down) DEGs in bulk RNA-seq datasets (|fold change|≥ 2, false discovery rate (FDR) < 0.05). Furthermore, gene ontology (GO) enrichment of these upregulated and downregulated genes revealed activated pathways that are associated with mitochondria damage and energy metabolism process, such as regulation of membrane potential, cellular carbohydrate, and carbohydrate biosynthetic process metabolic process, and polysaccharide metabolic process (Fig. 8). The bulk transcriptomics analysis results indicate that TPP-SS-ATS-LS can target mitochondria and affect mitochondrial function, which is consistent with our expectations. We also observed the down-regulation of epithelial (cancer) cell proliferation in the TPP-SS-ATS-LS group compared with the prototype drug ATS group (Fig. 8D), just as it was designed.

### The suppression of mitochondrial respiration in breast carcinoma

**Fig. 9:**
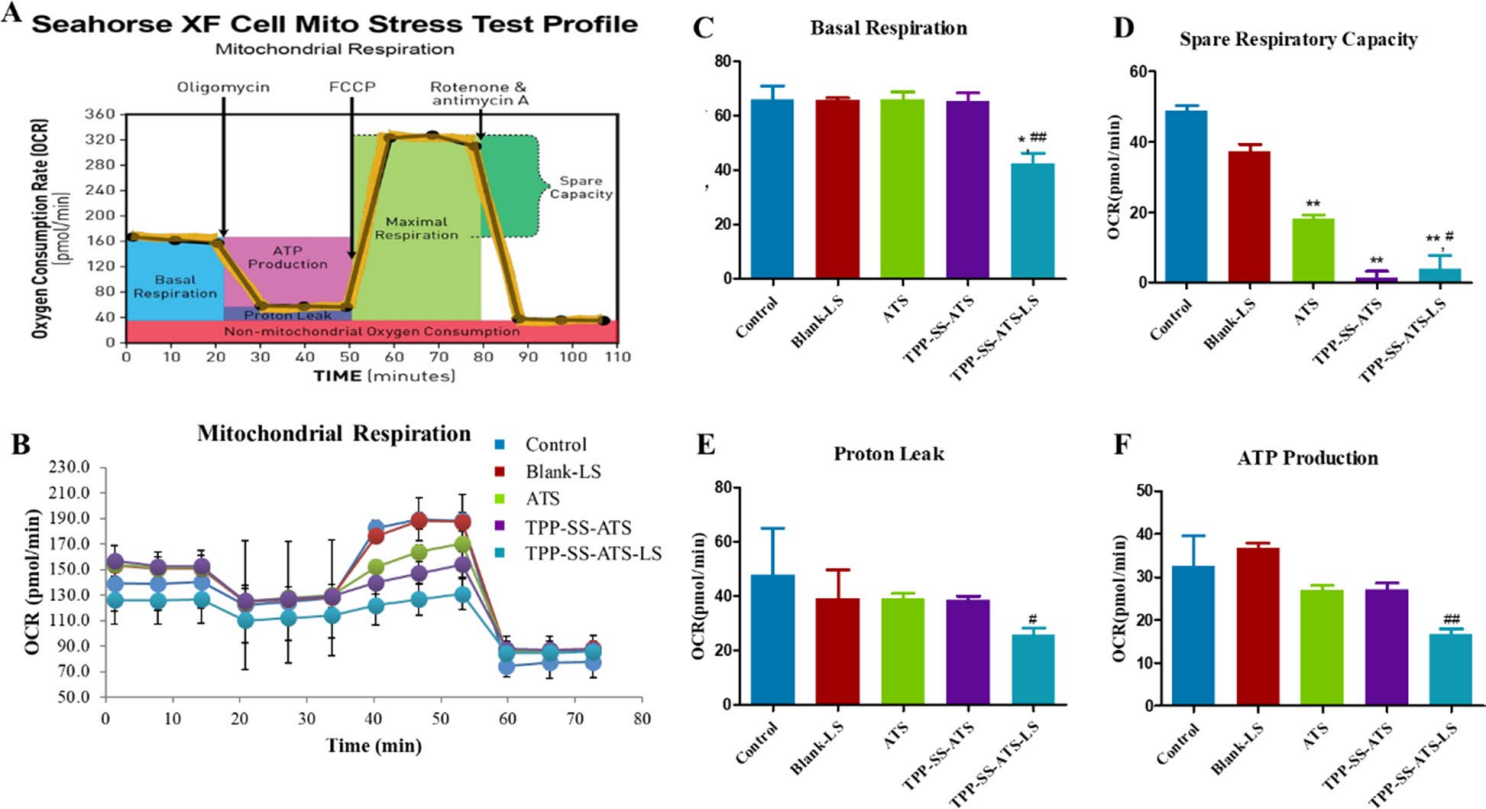
Effect of TPP-SS-ATS-LS on oxygen consumption rate (OCR) of breast MDA-MB-231 cell. **A** A schematic overview of the mitochondrial stress test. The black arrows indicate the subsequent addition of the ATPase inhibitor oligomycin, the uncoupling reagent FCCP, and the inhibitors of the electron transport chain rotenone/antimycin A. **B** MDA-MB-231 cells were seeded in a 96-well Seahorse cell culture plate overnight (mean ± SD, n = 3). Incubation with blank-LS, ATS, TPP-SS-ATS or TPP-SS-ATS-LS at concentration of 20 µM for 24 h (**C**–**F**). These are respectively representing individual parameters for (**C**) basal respiration. **D** Spare respiration, (**E**) proton Leak and (**F**) ATP linked respiration. Compared with Control group, *P < 0.05, **P < 0.01. Compared with ATS group, ^#^*P* < 0.05, ^##^*P* < 0.01

To effectively examine the metabolic and bioenergetic functions of metabolic activity alterations induced by TPP-SS-ATS-LS, the basal and maximal respiration, oxygen consumption rate (OCR) was assessed using Seahorse XF96 extracellular flux analyzer using the Cell Mito Stress Test Kit(Fig. 9). As shown in Fig. 9B, OCR parameters including the mitochondrial function and capacity decreased in a concentration-dependent manner in breast carcinoma cells following TPP-SS-ATS-LS administration compared with the control group. The basal respiration (Fig. 9C) spare respiration (Fig. 9D), proton leak (Fig. 9E) and ATP linked respiration (Fig. 9F) of tumor cells were significantly suppressed. Altogether, these results clearly indicated that TPP-SS-ATS-LS could almost completely suppress mitochondrial basal and maximal respiration in all 3 breast carcinoma cells in vitro (shown in Additional file 1: Figs. S14, S15).

### TPP-SS-ATS-LS induced mitophagy through inhibiting PHB2 expression and upregulating LC3-II and PINK1 protein expression

**Fig. 10:**
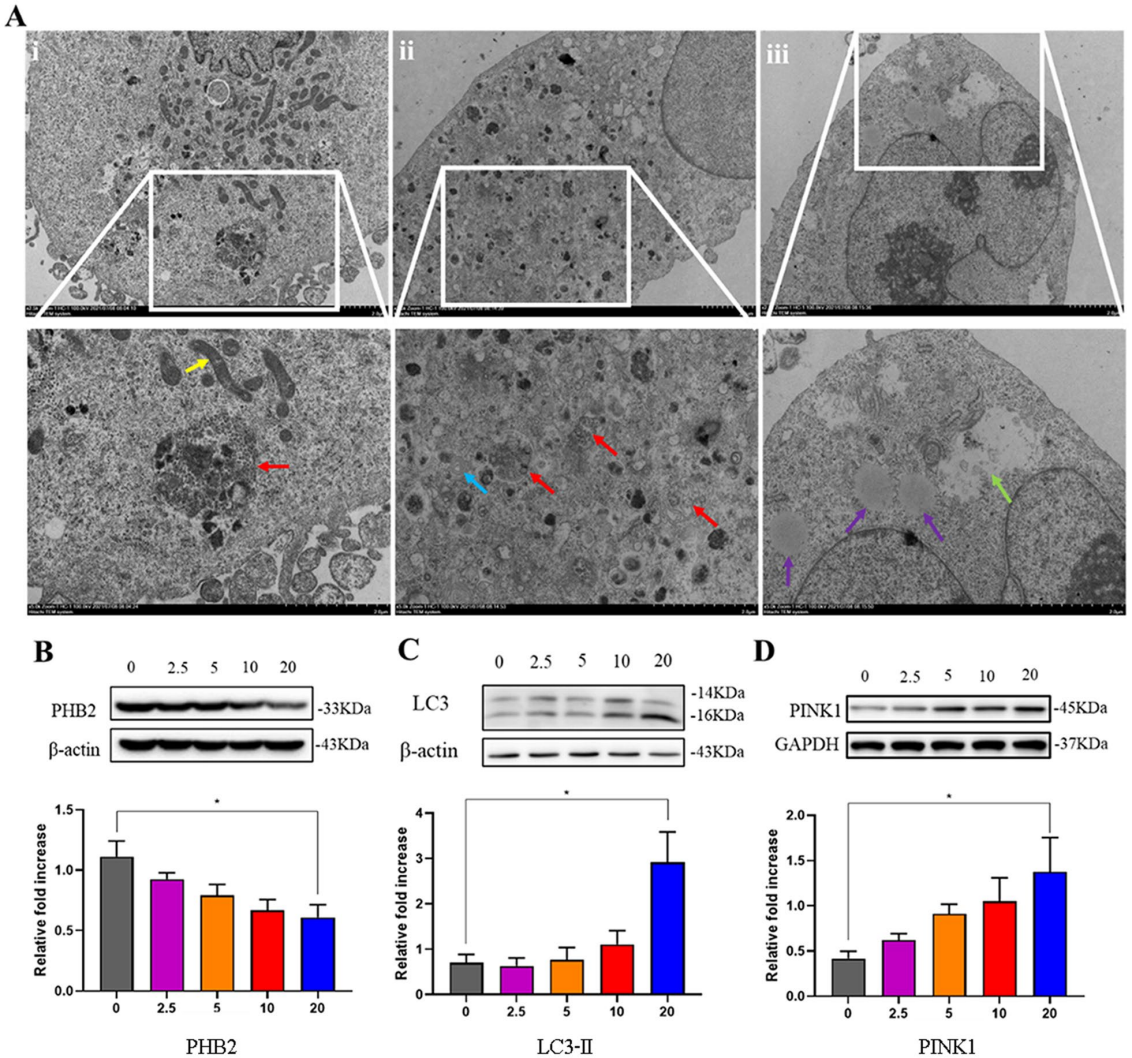
Induced autophagy of breast cancer cell. **A** TEM results of MDA-MB-231cells after 20 µM TPP-SS-ATS-LS treatment. (i) Control group, (ii) and (iii) TPP-SS-ATS-LS group. The mitochondria were shown by the yellow arrows; the autophagy lysosomes shown in red arrows, the myelin-like structures shown by the blue arrows. Local organelles self-melt and degrade as shown by the green arrows, and individual lipids are shown by the purple arrows. The protein expression level was analyzed in MDA-MB-231 cells after TPP-SS-ATS-LS treatment at 0, 2.5, 5, 10 and 20 µM for 48 h by Western blot analysis. TPP-SS-ATS-LS down-regulated (**B**) PHB2 protein level and induced (**C**) LC3-II and (**D**) PINK1 protein expression increased in breast cancer cells. Data are presented as the mean ± SD of at least three independent experiments. Compared with Control group, *P < 0.05

The underlying mechanism for the enhanced therapeutic effects of TPP-SS-ATS-LS was investigated by transmission electron microscope imaging in cultured cells and western blot analysis. As shown in Fig. 10A, mitophagy could be clearly observed in MDA-MB-231cells after 20 µM TPP-SS-ATS-LS treatment. In the control group (i): the number of mitochondria was abundant in the visual field, as shown by the yellow arrow. There is a small amount of autophagolysosomes (indicated by the red arrow) that could be observed in the control group. Compared with the control group, there were no obvious mitochondria in the visual field, and the number of mitochondria was significantly reduced in TPP-SS-ATS-LS group (ii) and (iii); The number of lysosomes increased significantly, most of the mitochondrial structures were unclear, and the number of mitochondrial cristae was reduced (indicated by the red arrows). Numerous myelin-like structures are seen in the cytoplasm, as shown by the blue arrow. Local organelles autolytic and degrade, as shown by the green arrow. The above results demonstrated that the treatment induced the appearance of autophagy.

PINK1 (PTEN induced putative kinase 1) mediated mitophagy is the main way to selectively scavenge damaged mitochondria, which has attracted much attention because of its important role in maintaining mitochondrial homeostasis. Studies have shown that PINK1 plays an important role in mitophagy in breast cancer cells [20, 48]. It acts as a gatekeeper of mitochondria and can perceive the presence of healthy or damaged mitochondria [49]. In healthy conditions PINK1 proteins continue to degrade and it keeps at a low level, whereas when mitochondria are damaged, PINK stabilizes and recruits the E3 ligase Parkin to initiate autophagy [50].

We have demonstrated that TPP-SS-ATS-LS induced mitophagy in mice tumor tissues through down-regulated PHB2 protein expression. Whether it affects PINK1 mediated mitophagy is worthy of researching. We evaluated the underlying mechanism in vitro*.* As presented in Fig. 10B, TPP-SS-ATS-LS blocked PHB2 protein expression in a dose-dependent manner compared with the control group in MDA-MB-231 cells, indicating that TPP-SS-ATS-LS inhibited MDA-MB-231 cells proliferation might be partially responsible for blocking protein expression of PHB2. We thus evaluated the effects of TPP-SS-ATS-LS treatment on the expression of LC3-II and PINK1 in cultured cells. As shown in Fig. 10C, D, TPP-SS-ATS-LS induced LC3-II and PINK1 protein expression (*P* < 0.05).

In this study, we provide the first evidence that the anti-tumor effect of TPP-SS-ATS-LS is the regulation of PINK1 dependent mitophagy probably connected with downregulating the expression of PHB2. It is known that PHB2 serves as an essential inner mitochondrial membrane mitophagy receptor in cancer and promotes PINK1-dependent mitophagy [22, 51]. Indeed, most breast cancer cells overexpress PHB2, which promotes resistance to chemotherapy-induced cell death [52, 53]. We found that TPP-SS-ATS-LS induced mitophagy in MDA-MB-231 cells, which was characterized by the decrease of PHB2 activation and enhanced LC3-II and PINK1 protein expression.

## Conclusion

Due to the lack of targeting ability, artesunate has limited anti-tumor efficacy. GSH sensitive artesunate conjugate (TPP-SS-ATS) with mitochondrial targeting function was synthesized in this study. TPP-SS-ATS was further encapsulated in liposomes (TPP-SS-ATS-LS) with tumor targeting function, which realized the step-by-step targeted delivery of artesunate to tumor cell and mitochondria, so as to improve the induction effect of artesunate on mitophagy. The superiorities of TPP-SS-ATS-LS against breast tumor were verified by in vivo and in vitro evaluations. The smart liposomes were firstly observed in tumor cells after administrating 4 to 6 h. Meanwhile, the hematological, blood biochemical and histological side effects were scarcely observed in treatment of tumor bearing mice. Through the pilot study on the antitumor mechanism, we found that TPP-SS-ATS-LS inhibit tumor cell proliferation through PINK1 dependent mitophagy, which was mediated by down-regulating the expression of PHB2. In-depth investigation of the anticancer properties of these constructs would be a key next step that could push forward new mechanistic exploration and reinforce the efficacy of artemisinin-like drugs. This paper provides new research strategies for the development of new artemisinin-based antitumor drugs.

## Supplementary Information

**Additional file 1**: **Fig. S1. **Chemical structure of SS-ATS and number of carbon atoms in SS-ATS. **Fig. S2. **Chemical structure of TPP-SS-ATS and number of carbon atoms in TPP-SS-ATS. **Fig. S3. **Mass spectrum of SS-ATS. **Fig. S4. **Mass spectrum of TPP-SS-ATS. **Fig. S5. **^1^H NMR spectrum of SS-ATS. **Fig. S6. **^1^H NMR spectrum of TPP-SS-ATS. **Fig. S7. **^13^C NMR spectrum of TPP-SS-ATS. **Fig. S8. **HSQC spectrum of TPP-SS-ATS. **Fig. S9. **HMBC spectrum of TPP-SS-ATS. **Fig. S10.** Purity check of TPP-SS-ATS chromatogram. **Fig. S11.** Ultraviolet spectrogram of TPP-SS-ATS. **Fig. S12. **Molecular dynamics simulation. **Fig. S13.** The hemolytic results of TPP-SS-ATS-LS. **Fig. S14.** Effect of TPP-SS-ATS -LS on oxygen consumption rate (OCR) of human breast MCF-7 cells. **Fig. S15.** Effect of TPP-SS-ATS-LS on oxygen consumption rate (OCR) of mouse breast 4T1 cells. **Table S1**. Result of TPP-SS-ATS purity check.

## Data Availability

All data are included in this published article.
