# Supplementary material for: Development of artesunate intelligent prodrug liposomes based on mitochondrial targeting strategy
Source: J Nanobiotechnology. 2022 Aug 13;20:376. doi: 10.1186/s12951-022-01569-5 (PMC9375379; doi:10.1186/s12951-022-01569-5)
Supplement: Supplementary file 1 — Additional file 1: Fig. S1. Chemical structure of SS-ATS and number of carbon atoms in SS-ATS. Fig. S2. Chemical structure of TPP-SS-ATS and number of carbon atoms in TPP-SS-ATS. Fig. S3. Mass spectrum of SS-ATS. Fig. S4. Mass spectrum of TPP-SS-ATS. Fig. S5. 1H NMR spectrum of SS-ATS. Fig. S6. 1H NMR spectrum of TPP-SS-ATS. Fig. S7. 13C NMR spectrum of TPP-SS-ATS. Fig. S8. HSQC spectrum of TPP-SS-ATS. Fig. S9. HMBC spectrum of TPP-SS-ATS. Fig. S10. Purity check of TPP-SS-ATS chromatogram. Fig. S11. Ultraviolet spectrogram of TPP-SS-ATS. Fig. S12. Molecular dynamics simulation. Fig. S13. The hemolytic results of TPP-SS-ATS-LS. Fig. S14. Effect of TPP-SS-ATS -LS on oxygen consumption rate (OCR) of human breast MCF-7 cells. Fig. S15. Effect of TPP-SS-ATS-LS on oxygen consumption rate (OCR) of mouse breast 4T1 cells. Table S1. Result of TPP-SS-ATS purity check. [file 12951_2022_1569_MOESM1_ESM.docx]

**Supporting Information**

**The Supplementary Material includes:**

**Supplement Figures and Table**

**Figure S1.** Chemical structure of SS-ATS and number of carbon atoms in SS-ATS.

**Figure S2.** Chemical structure of TPP-SS-ATS and number of carbon atoms in TPP-SS-ATS.

**Figure S3.** Mass spectrum of SS-ATS.

**Figure S4.** Mass spectrum of TPP-SS-ATS.

**Figure S5.** ^1^H NMR spectrum of SS-ATS.

**Figure S6.** ^1^H NMR spectrum of TPP-SS-ATS.

**Figure S7.** ^13^C NMR spectrum of TPP-SS-ATS.

**Figure S8.** HSQC spectrum of TPP-SS-ATS.

**Figure S9.** HMBC spectrum of TPP-SS-ATS.

**Figure S10.** Purity check of TPP-SS-ATS chromatogram.

**Figure S11.** Ultraviolet spectrogram of TPP-SS-ATS.

**Figure S12.** Molecular dynamics simulation.

**Figure S13.** The hemolytic results of TPP-SS-ATS-LS.

**Figure S14.** Effect of TPP-SS-ATS-LS on oxygen consumption rate (OCR) of human breast MCF-7 cells.

**Figure S15.** Effect of TPP-SS-ATS-LS on oxygen consumption rate (OCR) of mouse breast 4T1 cells.

**Table S1** Result of TPP-SS-ATS purity check.

**Supporting Information**

**Supplement Figures and Table**

**Figure S1.** Chemical structure of SS-ATS and number of carbon atoms in SS-ATS.

**Figure S2.** Chemical structure of TPP-SS-ATS and number of carbon atoms in TPP-SS-ATS.


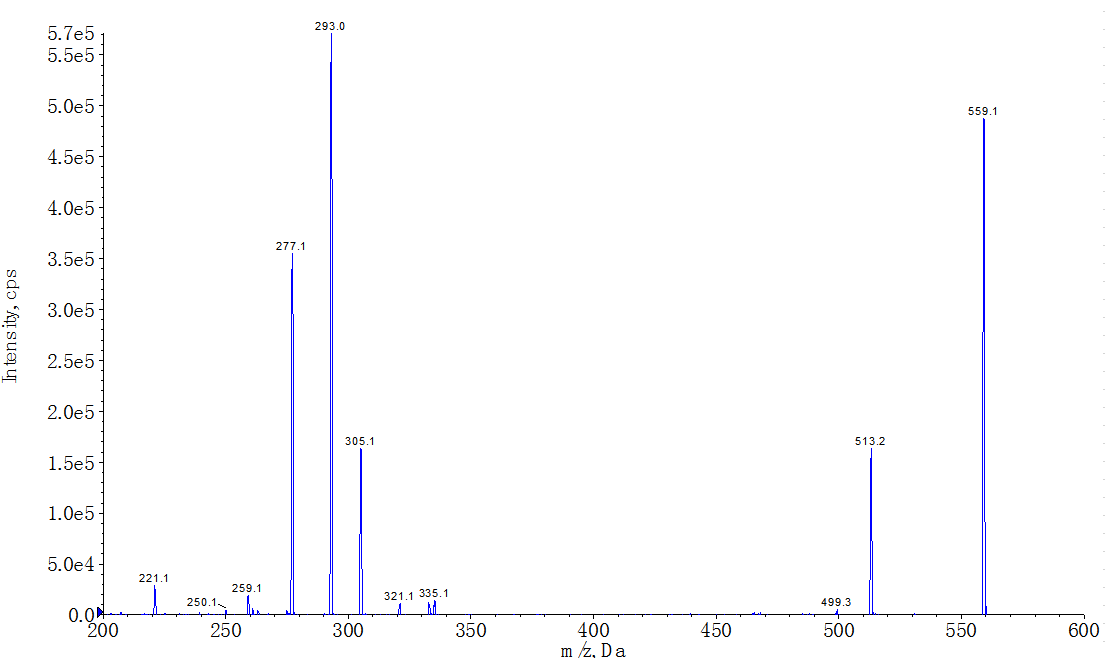


**Figure S3.** Mass spectrum of SS-ATS.


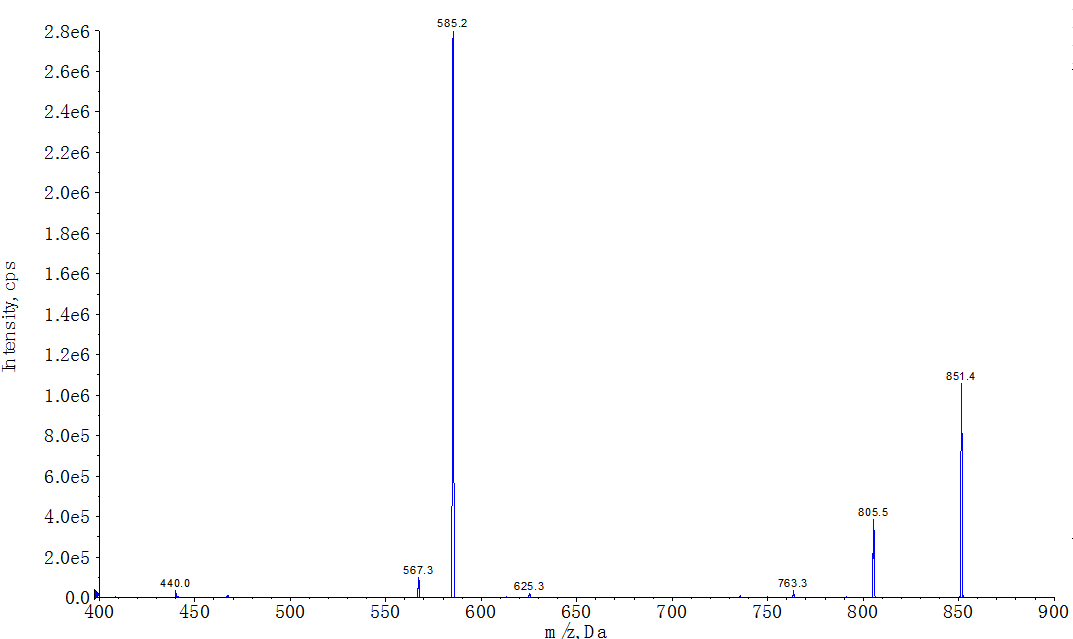


**Figure S4.** Mass spectrum of TPP-SS-ATS.

**
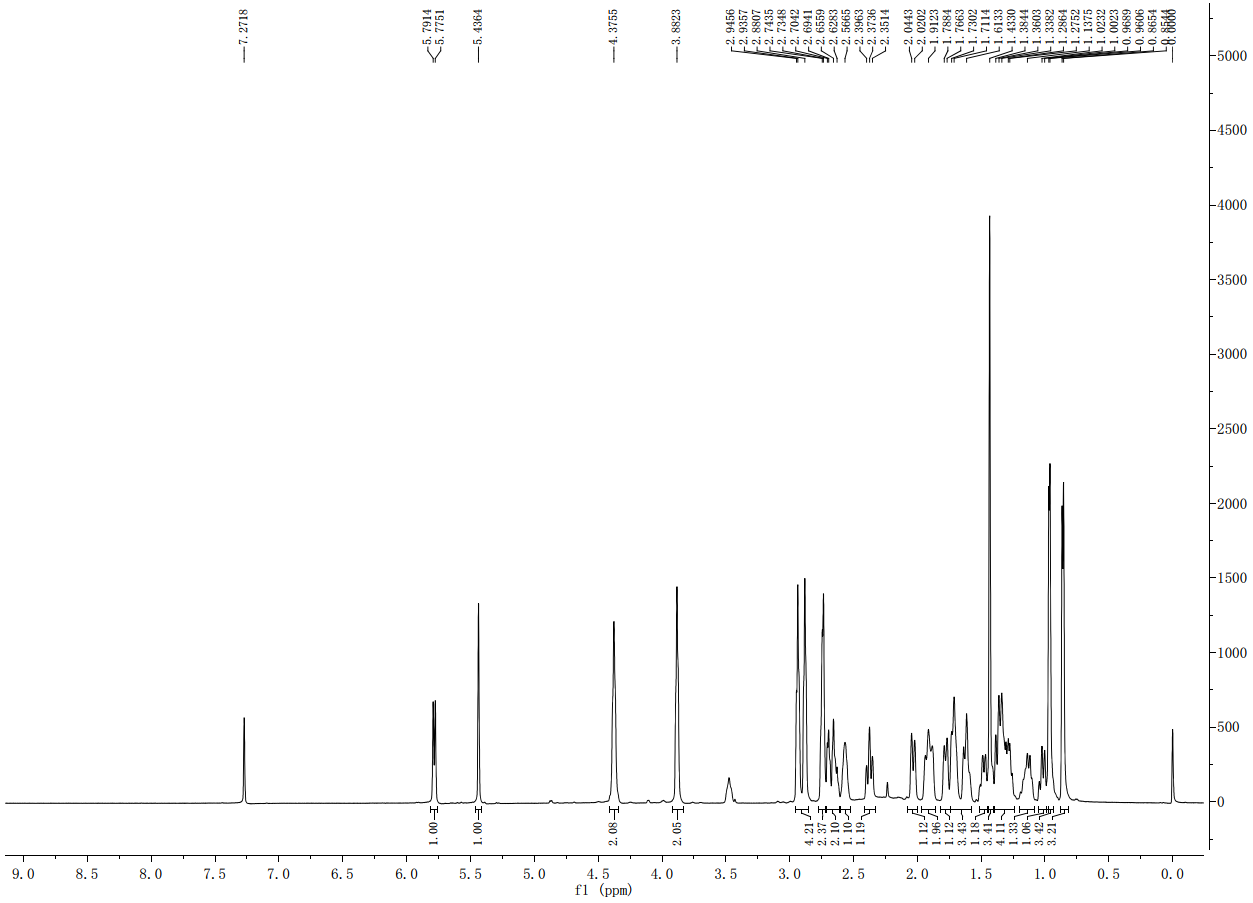
**

**Figure S5.** ^1^H NMR spectrum of SS-ATS.

**
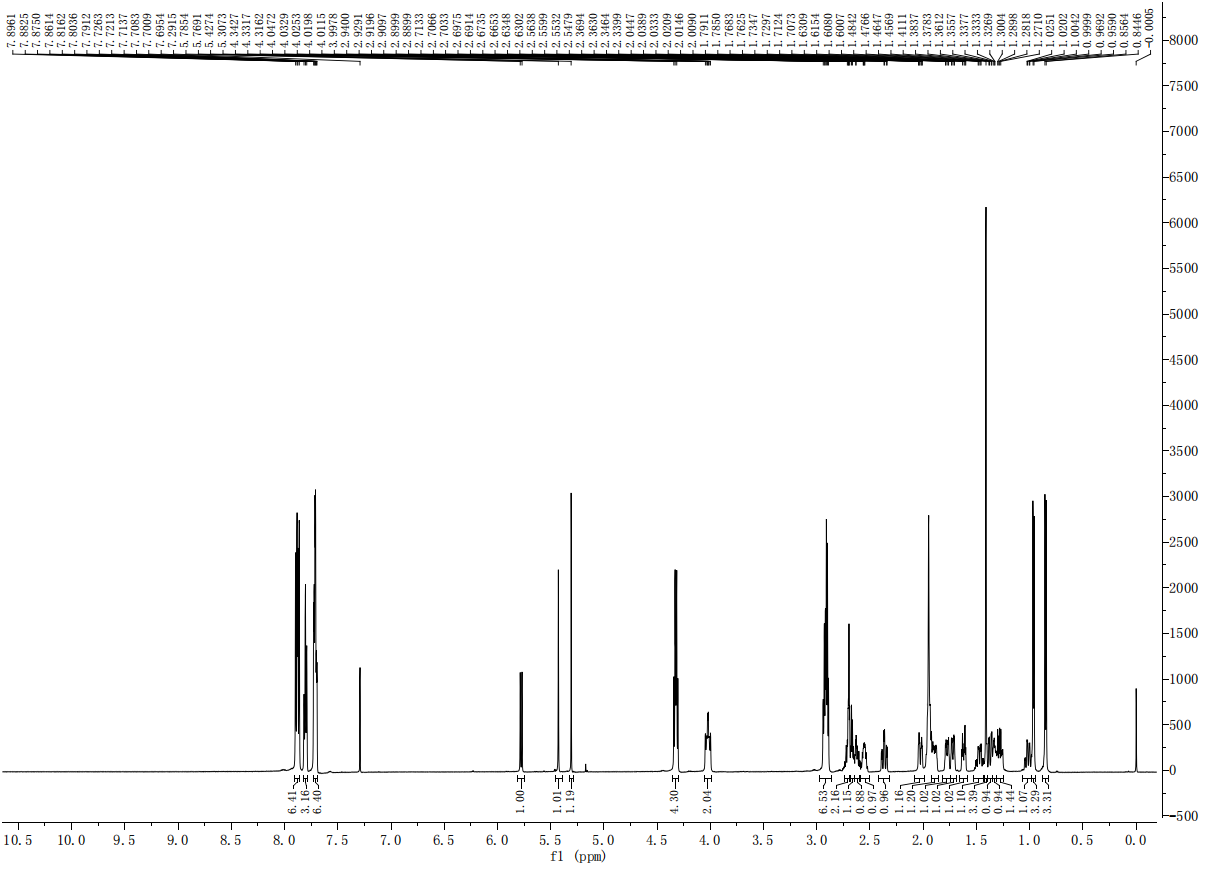
**

**Figure S6.** ^1^H NMR spectrum of TPP-SS-ATS.

**
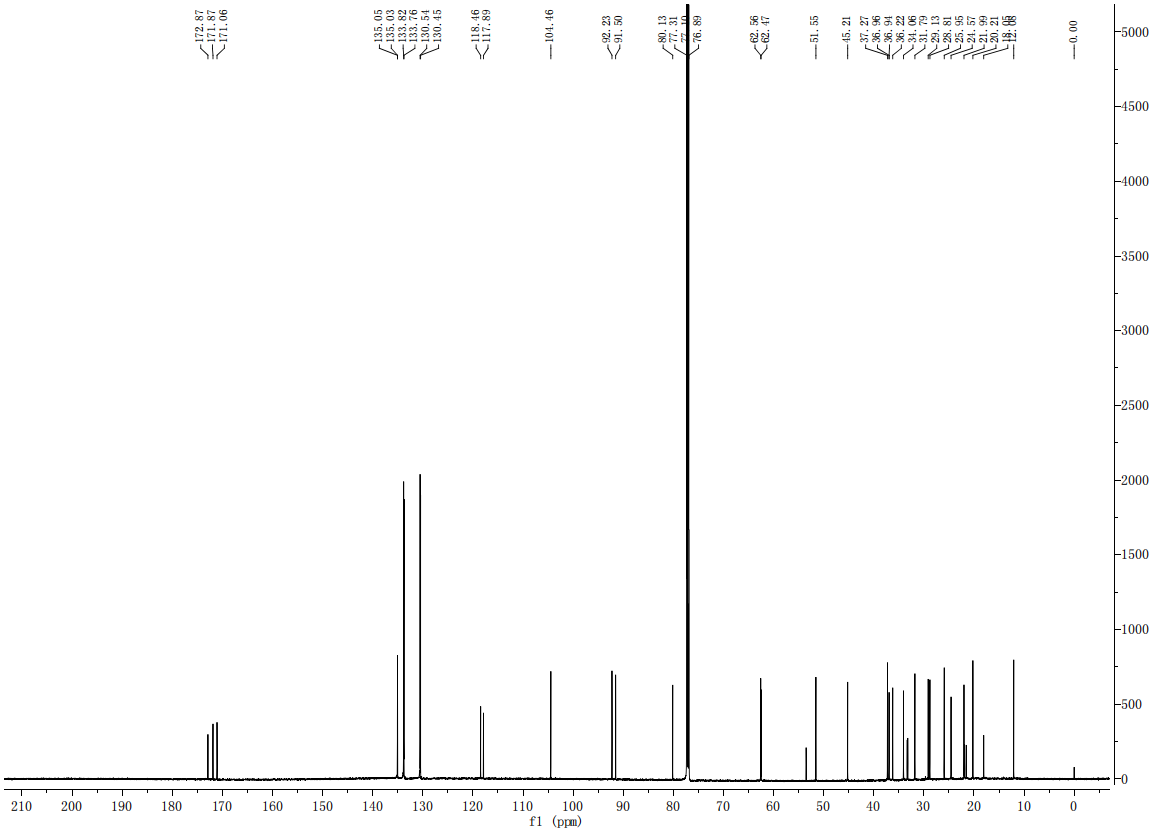
**

**Figure S7.** ^13^C NMR spectrum of TPP-SS-ATS.

**
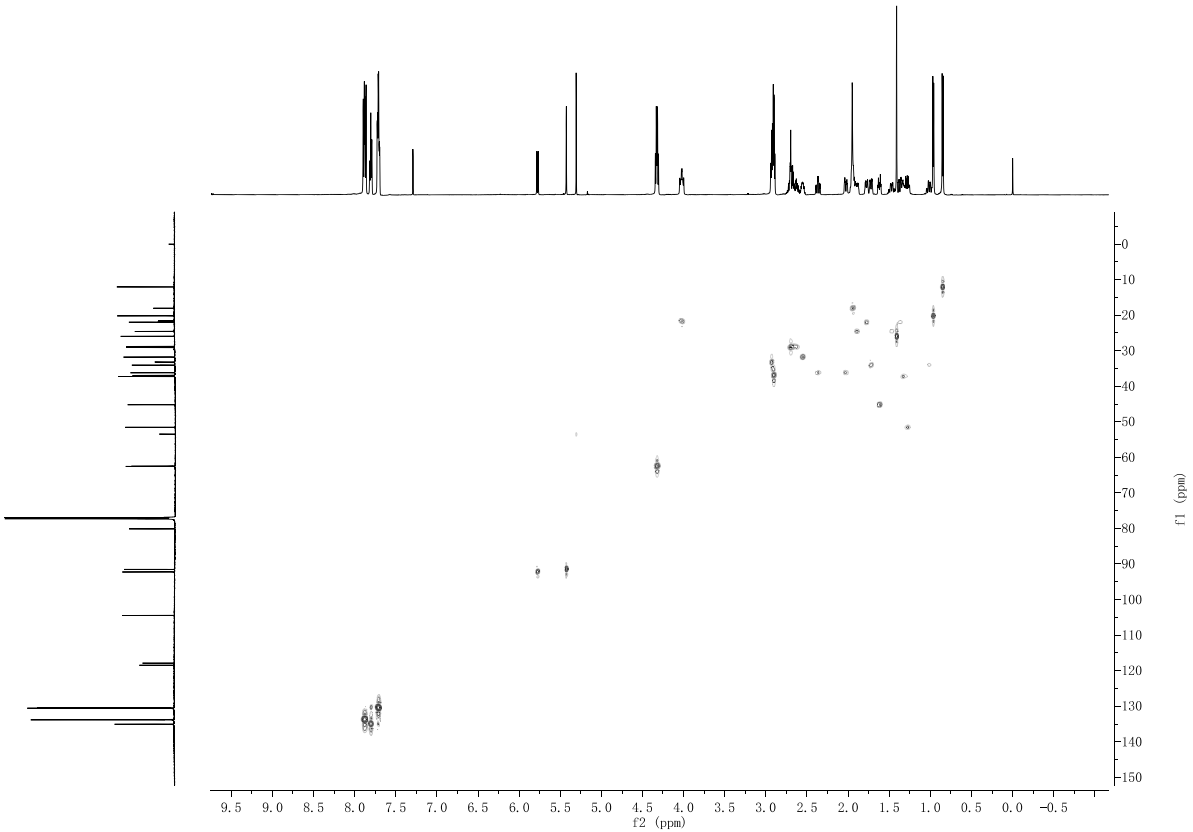
**

**Figure S8.** HSQC spectrum of TPP-SS-ATS.

**
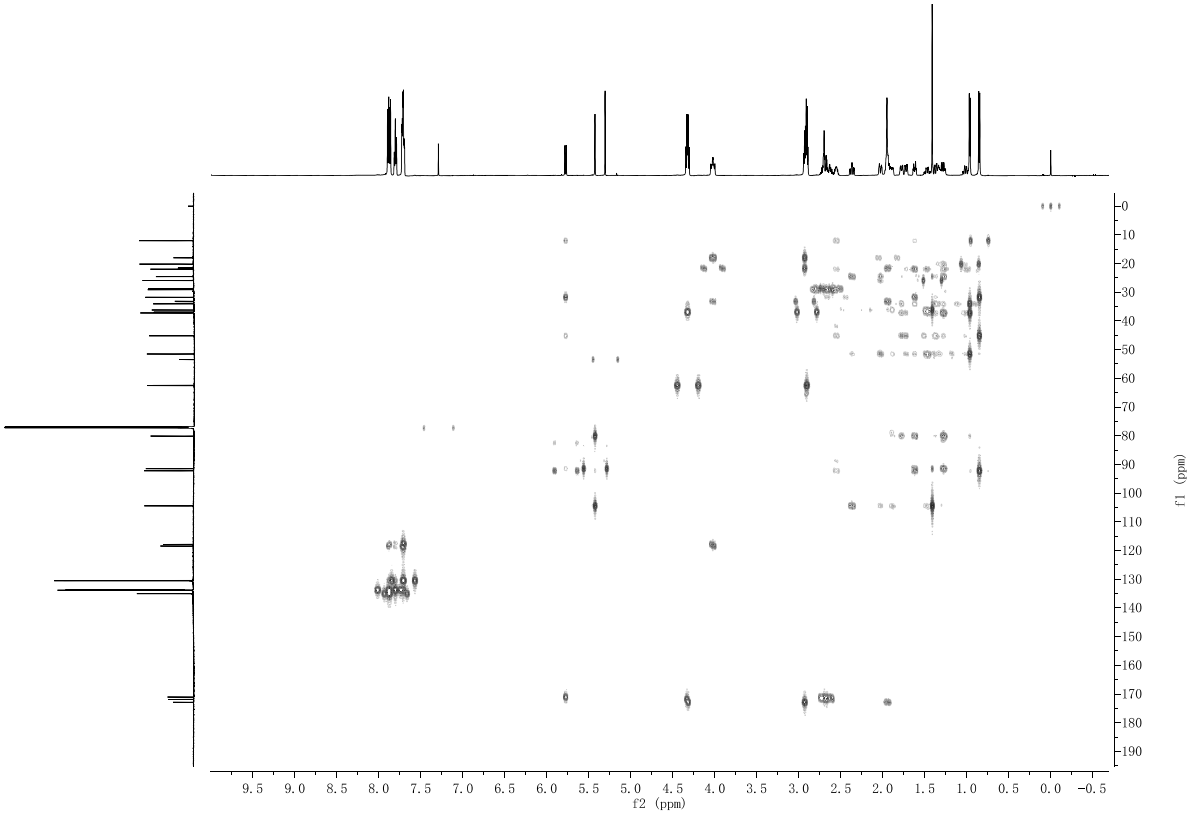
**

**Figure S9.** HMBC spectrum of TPP-SS-ATS.

**Figure S10.** Purity check of TPP-SS-ATS chromatogram.

**Figure S11** Ultraviolet spectrogram of TPP-SS-ATS.

**
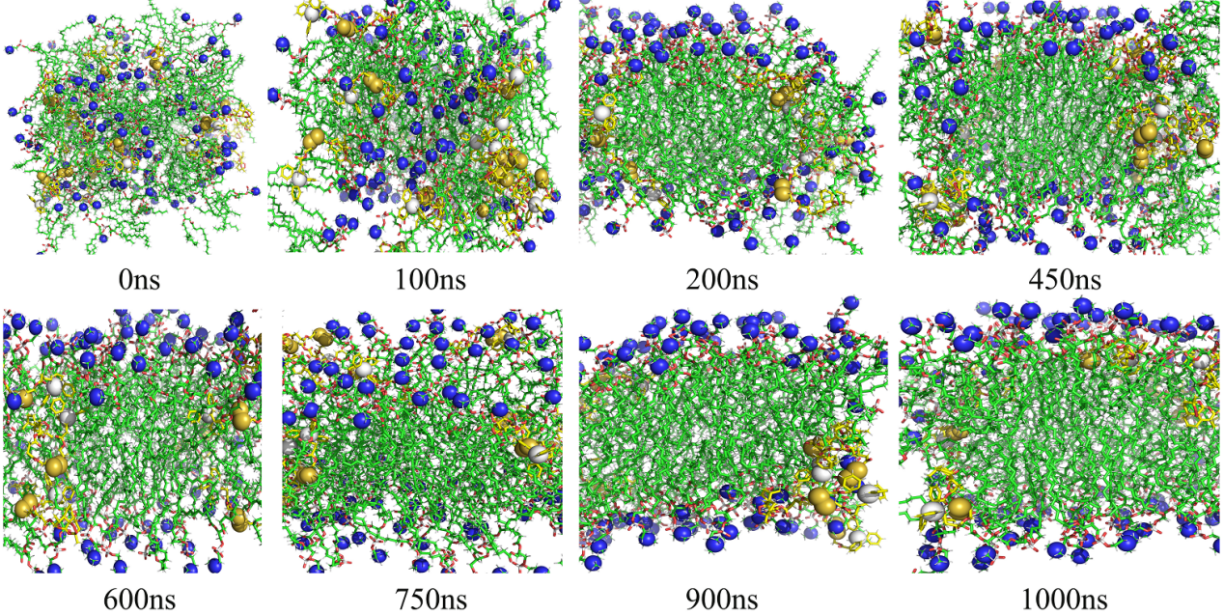
**

**Figure S12.** Molecular dynamics simulation: the formation process of liposome membrane structure. Blue marks are nitrogen atoms, yellow marks are sulfur atoms, and gray marks are phosphorous atoms. Note: water molecules are not shown in the figure.

**
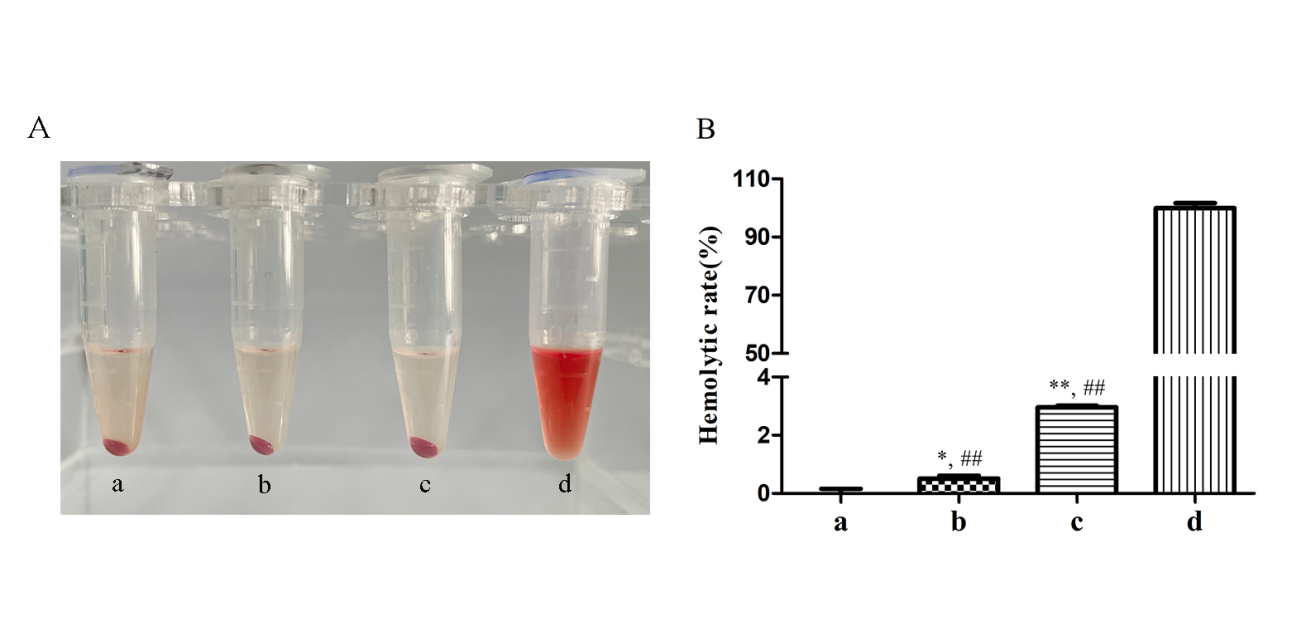
**

**Figure S13.** The hemolytic results of TPP-SS-ATS-LS. (A)The dosage of ***hemolytic assay*** was the same as in vivo anti-tumor experiments. (B) Hemolytic rate of TPP-SS-ATS-LS. a, PBS; b, TPP-SS-ATS-LS-L; c, TPP-SS-ATS-LS-H and d, 1%Triton-100-PBS positive control. Compared with PBS control group, **, P* < 0.05, ***, P* < 0.01. Compared with positive control group, ^#^*, P* < 0.05, ^##^*, P* < 0.01.


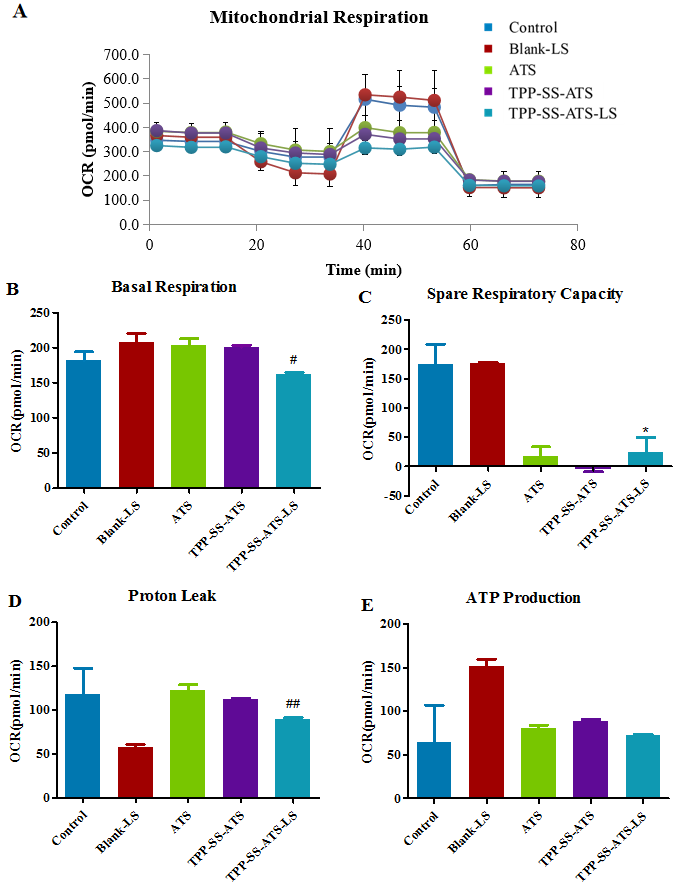


**Figure S14.** Effect of TPP-SS-ATS-LS on oxygen consumption rate (OCR) of human breast MCF-7 cells. (A) Mitochondrial stress test of MCF-7 cells after incubation with blank-LS, ATS, TPP-SS-ATS or TPP-SS-ATS-LS at concentration of 20 µM for 24 h. These are respectively representing individual parameters for (C) basal respiration; (D) spare respiration, (E) proton Leak and (F) ATP linked respiration. Compared with Control group, *, *P*< 0.05, **, *P* < 0.01. Compared with ATS group, ^#^, *P* < 0.05, ^##^, *P* < 0.01.


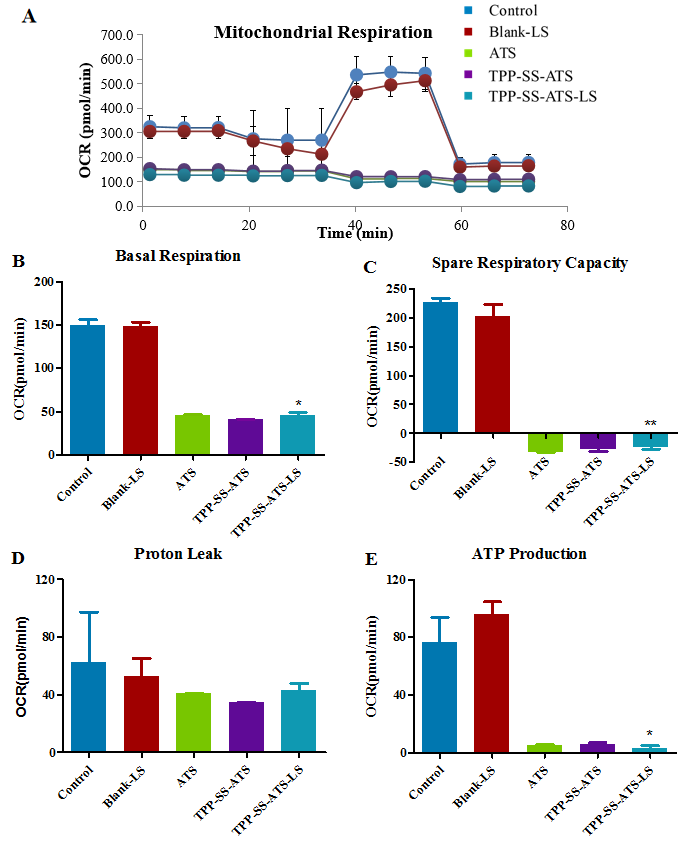


**Figure S15.** Effect of TPP-SS-ATS-LS on oxygen consumption rate (OCR) of mouse breast 4T1 cells. (A) Mitochondrial stress test of 4T1 after incubation with blank-LS, ATS, TPP-SS-ATS or TPP-SS-ATS-LS at concentration of 20 µM for 24 h. These are respectively representing individual parameters for (C) basal respiration; (D) spare respiration, (E) proton Leak and (F) ATP linked respiration. Compared with Control group, *, *P*< 0.05, **, *P* < 0.01.

**Table S1** Result of TPP-SS-ATS purity check

| No. | peak | retention time (min) | peak area | % peak area |
| --- | --- | --- | --- | --- |
| 1 | 1 | 3.371 | 2466 | 0.30 |
| 2 | 2 | 7.515 | 4698 | 0.57 |
| 3 | TPP-SS-ATS | 16.940 | 795548 | 97.25 |
| 4 | 3 | 17.414 | 4478 | 0.55 |
| 5 | 4 | 22.389 | 5356 | 0.65 |
| 6 | 5 | 22.822 | 5514 | 0.67 |
